# Frailty and prefrailty are associated with increased risk of aortic aneurysm but not aortic dissection: a prospective cohort study of UK biobank participants

**DOI:** 10.1186/s12872-026-06175-z

**Published:** 2026-07-01

**Authors:** Miaomiao Yang, Jianwu Zhang, Jiajing Chen, Yintong Teng, Xingsheng Ye, Qiguang Li, Lirong Zhang, Kun Zhang, Yuegang Wang, Aimin Dang, Weijing Feng

**Affiliations:** 1 Department of Special Care Center, National Clinical Research Center for Cardiovascular Diseases, Fuwai Hospital, Chinese Academy of Medical Sciences and Peking Union Medical College, No. 167 Bei Li Shi Road, Xi Cheng District, Beijing, 100037 China; 2 Department of Cardiology, State Key Laboratory of Organ Failure Research, Guangdong Provincial Key Lab of Cardiac Function and Microcirculation, Nanfang Hospital, The First School of Clinical Medicine, Southern Medical University, Guangzhou, 510515 China; 3 The Tenth Affiliated Hospital of Southern Medical University (Dongguan People’s Hospital), Southern Medical University or The First School of Clinical Medicine, Southern Medical University, Dongguan, 523018 China; 4 Department of Cardiology, Fuwai Hospital Chinese Academy of Medical Sciences Shenzhen, Shenzhen, Guangdong China; 5 Department of Cardiology, The Seventh Affiliated Hospital of Sun Yat-Sen University, 628 Zhenyuan Road, Shenzhen, 518107 China

**Keywords:** Frailty, Aortic aneurysm, Aortic dissection, Cohort study, Risk factor, Aging

## Abstract

**Background:**

Frailty is a well-established determinant of adverse outcomes in patients with aortic disease, but its role in incident aortic aneurysm (AA) and aortic dissection (AD) remains unclear. We examined the associations of frailty with long-term risks of AA and AD in a large community-based cohort.

**Methods:**

We included 474,302 UK Biobank participants free of AA/AD at baseline. Frailty was assessed using physical frailty and the frailty index, categorized as non-frail, prefrail, or frail. The primary outcomes were AA, with secondary outcomes, including abdominal AA (AAA), thoracic AA (TAA) and AD, ascertained through linkage to hospital and death records.

**Results:**

During a median follow-up of 15.1 years, 3,675 AA, 2,289 AAA, 1,114 TAA, and 310 AD events occurred. After multivariable adjustment, both prefrailty (physical frailty: HR 1.24, 95% CI 1.16–1.33; frailty index: HR 1.67, 95% CI 1.55–1.80) and frailty (physical frailty: HR 1.50, 95% CI 1.28–1.76; frailty index: HR 2.22, 95% CI 1.96–2.51) were associated with higher risk of AA versus non–frailty. Similar results were observed for AAA. For TAA, the frailty index remained significant (prefrailty: HR 1.47, 95% CI 1.29–1.67; frailty: HR 1.61, 95% CI 1.25–2.07), whereas physical frailty was not. Neither frailty measure was associated with AD. Subgroup analyses suggested stronger associations for AAA in younger participants and those without diabetes.

**Conclusions:**

Frailty was associated with a higher risk of incident AA, particularly AAA, but not AD. Frailty assessment may help improve phenotype-specific risk stratification and inform preventive strategies for aortic disease.

**Graphical Abstract:**

**Figure:**
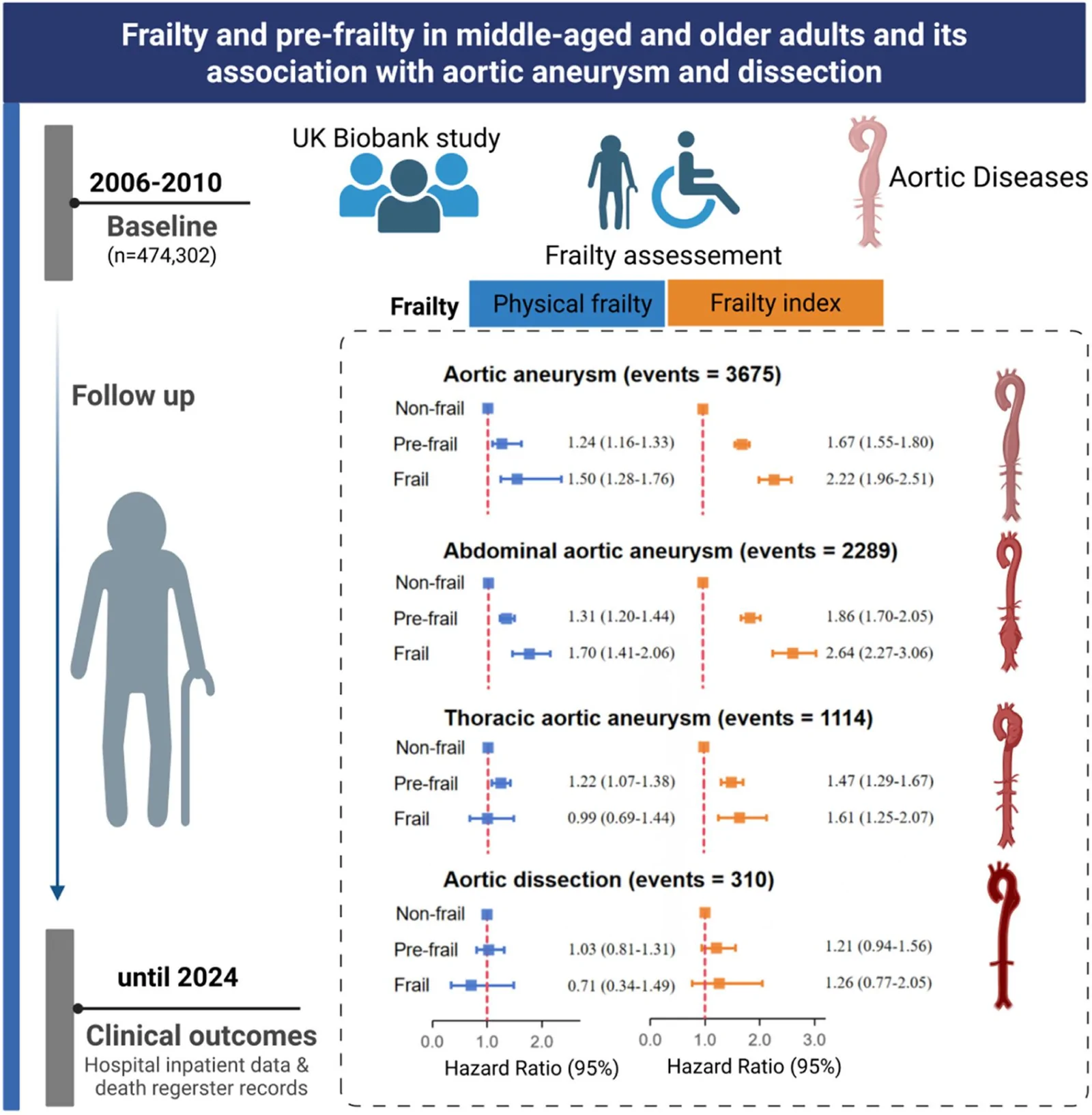

**Supplementary Information:**

The online version contains supplementary material available at https://doi.org/10.1186/s12872-026-06175-z.

## Background

Aortic aneurysm (AA) and aortic dissection (AD) represent a formidable threat to global cardiovascular health, with high mortality, substantial morbidity, and considerable economic costs [1–3]. Despite advances in diagnostic techniques and surgical interventions, the mortality rate associated with ruptured aneurysms remains alarmingly high, exceeding 50% [4]. In the case of untreated AD, fatality rates soar, with nearly 50% of patients succumbing within 48 h and 72% within a week [5]. Without timely intervention, both AA and AD are virtually 100% fatal [6]. These aortic pathologies, including thoracic aortic aneurysm (TAA) and abdominal aortic aneurysm (AAA), show a strong age-dependent prevalence, exceeding 8.3% in adults over 65 years of age [2, 3, 7]. Given the limited efficacy of current pharmacological interventions in halting the progression of AA or preventing AD, there is an urgent need to identify novel, modifiable risk factors that reflect underlying biological vulnerability and could inform early intervention strategies [2, 8]. 

Frailty, a multidimensional geriatric syndrome characterized by diminished physiological reserve and heightened vulnerability to adverse outcomes, has emerged as a key marker of biological aging and systemic functional decline [9, 10]. Frailty is associated with an elevated risk of various age-related conditions, including falls, disability, and all-cause mortality [11, 12]. While the relationship between frailty and cardiovascular diseases is well-documented [13, 14], its association with AA remains poorly understood. Notably, frail individuals experience a higher incidence of postoperative complications following aortic surgical repair, suggesting a potential underlying vulnerability of the aorta in frail individuals [15]. However, most existing research has focused on frailty as a prognostic factor in individuals with established aortic disease, with limited exploration into whether frailty is an early risk factor for incident AA and AD in the general population. Recent UK Biobank evidence has linked frailty to incident AAA [16]. However, that study focused specifically on AAA, and whether frailty is similarly associated with other aortic phenotypes, particularly TAA and AD, remains unclear. This distinction is clinically and biologically relevant because AAA, TAA, and AD differ in anatomical location, disease course, and underlying pathophysiology. Moreover, whether physical frailty and the deficit-accumulation frailty index show similar or divergent associations across these phenotypes has not been well characterized.

In this study, we aimed to examine whether frailty shows differential associations across incident aortic phenotypes in the general population. By leveraging longitudinal data from the UK Biobank, we sought to determine whether physical frailty and frailty index are independently associated with the development of aortic pathologies, including AAA, TAA, and AD, beyond traditional cardiovascular risk factors and chronological age. By focusing on phenotype-specific patterns rather than a single aortic endpoint, our study may offer further insight into the biological links between functional decline and aortic vulnerability, and may help inform risk stratification and preventive strategies for high-risk frail populations.

## Methods

### Study design and population

The UK Biobank (UKBB) is a large, ongoing population-based prospective cohort study that recruited over 500,000 community-dwelling participants aged 37–73 years, between 2006 and 2010 from 22 assessment centers across England, Scotland, and Wales [17]. At the baseline visit, comprehensive data were collected from each participant through questionnaires, verbal interviews, physical measurements, and biological samples. The study protocols were approved by the North West Multicenter Research Ethics Committee, and written informed consent was obtained from all participants. The UK Biobank resource is openly accessible to approved researchers via its official platform (https://www.ukbiobank.ac.uk/).

For the current analysis, among 502,137 participants who took part in the initial assessment visit from 2006 to 2010, those with AD/AA (*n* = 762) at baseline, as well as those with missing information on frailty measures (*n* = 27,073) were excluded. A total of 474,302 eligible participants were included in the final analysis.

### Exposure assessment

Frailty at baseline was evaluated using two complementary methodologies: physical frailty and the frailty index, which capture distinct dimensions of the frailty phenotype and offering clinically relevant insights from different perspectives [18]. 

In this study, physical frailty was evaluated using a modified version of the Fried frailty phenotype, adapted to the data available within the UK Biobank cohort [11, 19]. Physical frailty was defined based on five criteria: unintentional weight loss, self-reported exhaustion, low levels of physical activity, reduced gait speed, and diminished grip strength (Table S1). Participants were categorized as non-frail (meeting none of the criteria), prefrail (meeting one or two criteria), or frail (meeting three or more criteria), consistent with established classification thresholds [11, 19]. The frailty index was calculated using 49 self-reported items encompassing domains of general health, chronic conditions, physical functioning, and psychological well-being (Table S2) [20], following a previously validated approach within the UK Biobank cohort. For participants with fewer than 10 missing responses, the frailty index was calculated as the proportion of deficits present out of the total number of items, yielding a score ranging from 0 to 1. Based on established cutoffs, individuals were classified as non-frail (< 0.12), prefrail (0.12–0.24), or frail (> 0.24) [21, 22]. 

### Outcome assessment

The UK Biobank collected detailed medical history data through linkage with national hospital inpatient records and death registries. Diagnostic information was coded using the International Classification of Diseases, 10th Revision (ICD-10), while surgical interventions were classified according to the OPCS-4 coding system. In this study, AA, AAA, TAA and AD cases were ascertained based on previously validated definitions within the UK Biobank, incorporating the earliest recorded ICD-10-coded hospital diagnosis or death related to AA, as well as relevant OPCS-4-coded surgical procedures (Table S3) [23]. The full diagnostic and procedure code list is provided in Table S3, and detailed descriptions of individual ICD-10 and OPCS-4 codes are available through the UK Biobank coding resources. Briefly, the overall AA endpoint included ICD-10 codes I71.1–I71.9; TAA was defined using I71.1 and I71.2; AAA was defined using I71.3 and I71.4; and AD was defined using I71.0. These ICD-10 codes include both ruptured aneurysm codes and codes described as “without mention of rupture”; ruptured aneurysms were therefore included in the corresponding aneurysm endpoints but were not analyzed separately. Relevant OPCS-4 procedure codes were additionally used to capture aortic interventions. Because the ICD-10 and OPCS-4 codes do not directly classify aneurysms by symptomatic status, the outcomes represent clinically recognized and coded events captured through linked health records; undiagnosed or uncoded asymptomatic aneurysms could not be captured. Participants were followed from baseline enrollment until the first occurrence of AA/AD diagnosis, death, loss to follow-up, or the end of follow-up on 1 April 2024, whichever came first.

### Ascertainment of covariates

A comprehensive set of covariates was included to adjust for potential confounders, including: age (years), sex (male or female), body mass index (BMI, kg/m²), annual household income (<£31,000 or ≥£31,000), Townsend Deprivation Index (TDI), ethnicity (White European vs. non-White), educational attainment (degree-level vs. non-degree), employment status (employed, retired, or unemployed), alcohol consumption (never vs. former/current), smoking status (never vs. former/current), healthy diet score (0–5) [24], physical activity level (none, low, medium, high) [11], systolic blood pressure (SBP, mmHg), glycated hemoglobin (HbA1c, mmol/mol), lipid profile (including HDL, LDL, triglycerides, and total cholesterol, mmol/L), and medication use (antihypertensive, antihyperglycemic, and lipid-lowering agents; yes or no).

### Statistical analysis

Baseline characteristics were summarized according to frailty status (non-frail, prefrail, and frail) using means with standard deviations (SD) or medians with interquartile ranges (IQR) for continuous variables, and frequencies with percentages for categorical variables. Differences between groups were evaluated using analysis of variance (ANOVA) or the Kruskal-Wallis test for continuous variables and the chi-squared test for categorical variables.

Cumulative incidence curves were estimated using Kaplan-Meier survival analysis, stratified by frailty status (non-frail, prefrail, frail). Cox proportional hazards models were applied to estimate hazard ratios (HRs) and 95% confidence intervals (CIs) for the associations between frailty and the risk of AA, AAA, TAA, and AD. Frailty was examined both as a categorical variable (non-frail, prefrail, frail) and as a continuous measure-per 1-unit increase in physical frailty and per 0.1-unit increase in frailty index score. The proportional hazards assumption was evaluated using Schoenfeld residuals; for covariates violating the assumption (*P* < 0.05), time-dependent interaction terms were incorporated into the models. Time-to-event was defined as the duration from the landmark date to the earliest occurrence of the outcome event, death, loss to follow-up, or administrative censoring. To explore dose-response relationships between continuous frailty measures and incident outcome diseases, restricted cubic spline functions with three knots were fitted in fully adjusted Cox models.

Furthermore, subgroup analysis by age, sex, smoking status, and cardiometabolic conditions (hypertension, diabetes, and hypercholesterolemia) were conducted to explore effect modification. In addition, Sensitivity analyses were performed to test the robustness of our findings: (1) excluding participants with prior vascular disease, cerebral aneurysm, or connective tissue disease at baseline; (2) excluding participants diagnosed with AA/AD within 5 years of follow-up to minimize reverse causality; (3) applying multiple imputations for missing covariates; and (4) accounting for competing risks using Fine and Gray subdistribution hazards models, with all-cause mortality treated as a competing event. All analyses were conducted using R program (version: 4.2.2).

## Results

### Baseline characteristics

Table 1 describes the baseline characteristics of participants according to physical frailty, whereas the frailty index is shown in Table S4. During a median follow-up of 15.12 (IQR: 1.03) years, 3,675 participants developed AA, 2,289 developed AAA, 1,114 developed TAA and 310 developed AD. The overlap between physical frailty and the frailty index is shown in Table S5. The weighted kappa estimate was 0.282 (95% CI: 0.279–0.285), and the Spearman correlation between the two frailty measurements was 0.310. Of the 474,302 participants, 15,546 (3.28%) were considered frail in accordance with physical frailty, and 26,502 (5.59%) were considered frail in accordance with the frailty index. Across the cohort, frail individuals were more likely to be older, female, of non-White ethnicity, current or former smokers, and never drinkers; they were also more likely to have lower educational attainment, poorer employment status, greater socioeconomic deprivation, higher BMI, lower healthy diet scores, and elevated levels of SBP and HbA1c.

**Table 1:** Baseline characteristics of the participants in accordance with physical frailty

Baseline Characteristics	Physical frailty				
	Overall	Non-frail	Prefrail	Frail	*P* value
No. (%)	474,302	249,983(52.71)	208,773(44.02)	15,546(3.28)	< 0.001
Follow-up (years)	15.12(1.03)	15.17(1.00)	15.06(1.06)	15.05(1.19)	< 0.001
Age (years)	56.51(8.08)	55.62(8.09)	57.44(7.99)	58.36(7.51)	< 0.001
Female sex, *n* (%)	258,184(54.44)	128,775(51.51)	119,673(57.32)	9736(62.63)	< 0.001
Ethnicity, *n* (%)					< 0.001
White Europeans	430,487(90.76)	229,168(91.67)	187,757(89.93)	13,562(87.24)	
Non-White Europeans	42,301(8.92)	20,133(8.05)	20,267(9.71)	1901(12.23)	
Missing	1514(0.32)	682(0.27)	749(0.36)	83(0.53)	
BMI (kg/m2)	27.35(4.74)	26.58(4.18)	27.72(4.89)	30.20(6.13)	< 0.001
Employment, *n* (%)					< 0.001
Employment	254,002(53.55)	148,443(59.38)	101,468(48.60)	4091(26.32)	
Retired	144,337(30.43)	67,635(27.06)	71,167(34.09)	5535(35.60)	
Unemployment	71,812(15.14)	31,953(12.78)	34,148(16.36)	5711(36.74)	
Missing	4151(0.88)	1952(0.78)	1990(0.95)	209(1.34)	
Education, *n* (%)					< 0.001
Degree-level education	54,658(11.52)	31,724(12.69)	21,896(10.49)	1038(6.68)	
Non-college education	336,350(70.91)	185,080(74.04)	142,671(68.34)	8599(55.31)	
Missing	83,294(17.56)	33,179(13.27)	44,206(21.17)	5909(38.01)	
Alcohol consumption status, *n* (%)					< 0.001
Never drinkers	19,898(4.20)	7432(2.97)	10,898(5.22)	1568(10.09)	
Current or former drinkers	453,974(95.71)	242,425(96.98)	197,622(94.66)	13,927(89.59)	
Missing	430(0.09)	126(0.05)	253(0.12)	51(0.33)	
Tobacco consumption status, *n* (%)					< 0.001
Never smokers	259,468(54.71)	141,322(55.53)	111,151(53.24)	695(45.00)	
Current or former smokers	213,230(44.96)	108,019(43.21)	96,768(46.35)	8443(54.31)	
Missing	1604(0.34)	642(0.26)	854(0.41)	108(0.69)	
Healthy diet score, *n* (%)					< 0.001
0–1	49,823(10.50)	26,416(10.56)	21,558(10.33)	1849(11.89)	
2–3	301,306(63.53)	159,814(63.93)	131,522(63.00)	9970(64.13)	
4–5	123,173(25.97)	63,753(25.50)	55,693(26.68)	3727(23.97)	
Physical activity, *n* (%)					< 0.001
Never	30,975(6.53)	16,250(6.50)	13,732(6.58)	993(6.39)	
Low activity	17,845(3.76)	9287(3.72)	7968(3.82)	590(3.80)	
Medium activity	371,409(78.31)	196,012(78.41)	163,266(78.20)	12,131(78.03)	
High activity	47,339(9.98)	24,886(9.96)	20,840(9.98)	1613(10.38)	
Missing	6734(1.42)	3548(1.42)	2967(1.42)	219(1.41)	
TDI	-1.35(3.06)	-1.69(2.86)	-1.07(3.18)	0.39(3.56)	< 0.001
Family income, *n* (%)					< 0.001
<£31 000	194,099(40.92)	89,081(35.63)	95,710(45.84)	9308(59.87)	
≥£31 000	213,856(45.09)	130,438(52.18)	80,591(38.60)	2827(18.18)	
Missing	66,347(13.99)	30,464(12.19)	32,472(15.55)	3411(21.94)	
SBP (mmHg)	137.81(18.61)	138.13(18.69)	137.63(18.55)	137.08(18.69)	< 0.001
HbA1c (%)	5.44(0.61)	5.37(0.50)	5.50(0.67)	5.78(1.00)	< 0.001
HDL cholesterol (mmol/L)	1.45(0.38)	1.48(0.39)	1.44(0.38)	1.36(0.37)	< 0.001
LDL cholesterol (mmol/L)	3.56(0.87)	3.59(0.85)	3.55(0.88)	3.42(0.92)	< 0.001
Triglycerides (mmol/L)	1.74(1.02)	1.69(1.01)	1.76(1.03)	1.90(1.08)	< 0.001
Cholesterol (mmol/L)	5.70(1.14)	5.75(1.11)	5.68(1.16)	5.48(1.23)	< 0.001
Anti-hypertensive medication, *n* (%)	52,687(11.11)	23,058(9.22)	26,694(12.79)	2935(18.88)	< 0.001
Anti-hyperglycaemic medication, *n* (%)	16,247(3.43)	4142(1.66)	9970(4.78)	2135(13.73)	< 0.001
Lipid-lowering medication, *n* (%)	49,263(10.39)	21,826(8.73)	24,705(11.83)	2732(17.57)	< 0.001

*Abbreviations*: *BMI* body mass index, *TDI* Townsend deprivation index, *SBP* systolic blood pressure, *HbA1c* haemoglobin A1c

Levels of significance: *P* <0.05 (ANOVA, Kruskal–Wallis H, and chi-squared tests)

### Frailty and risks of incident AA and AD

**Fig. 1:**
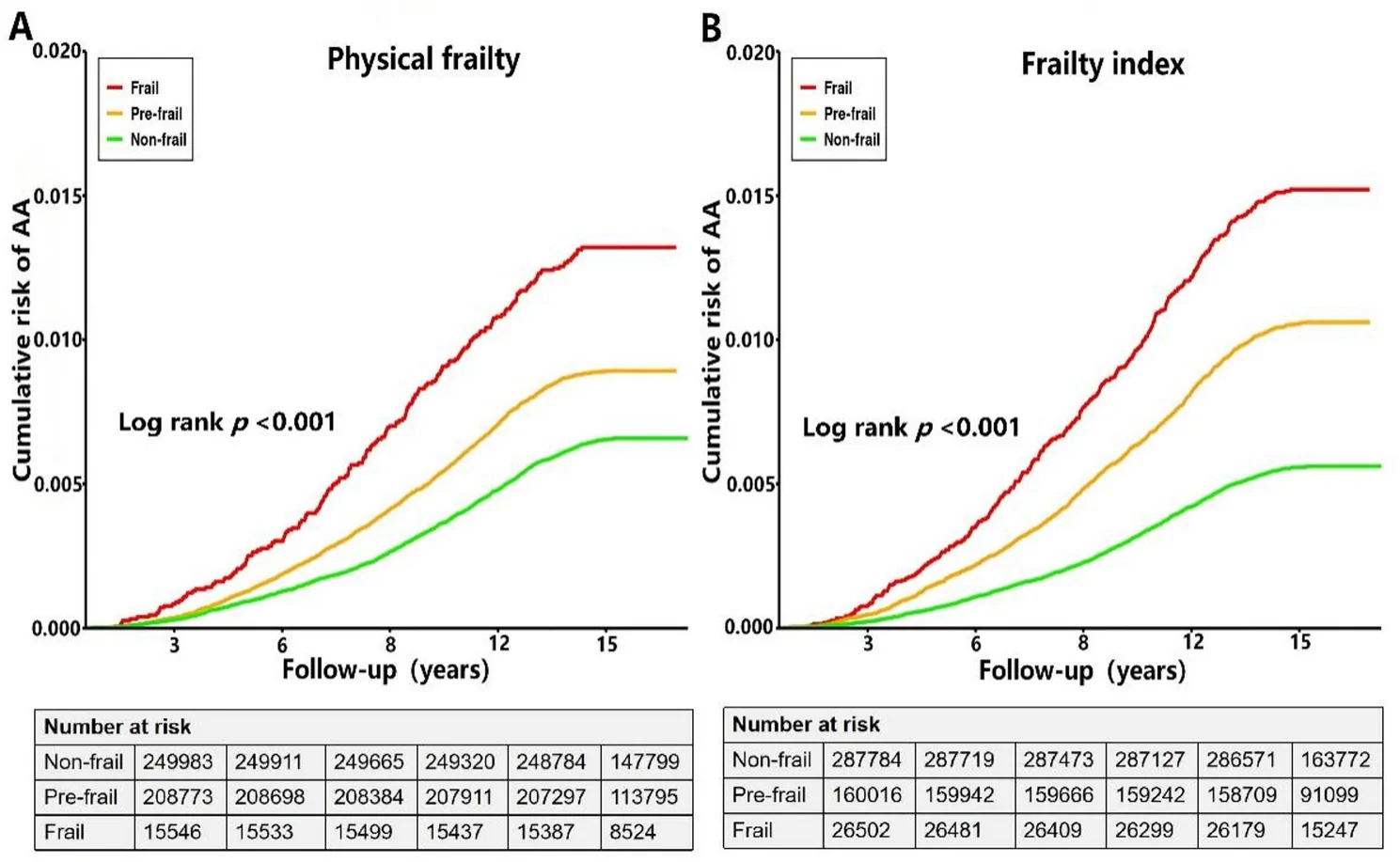
Cumulative risks of incident AA according to frailty profile. (A) Cumulative risks of incident AA according to physical frailty status. (B) Cumulative risks of incident AA according to frailty index status. Levels of significance: *P* < 0.05 (log-rank test). Abbreviations: AA, aortic aneurysm

The Kaplan-Meier curves indicated differences in cumulative risks for AA, AAA, TAA and AD across three frailty categories (Fig. 1 and Figure S1). In contrast to the significant differences observed for AA, AAA, and TAA (all log-rank *P* < 0.05), the cumulative incidence of AD did not vary significantly across frailty categories (log-rank *P* = 0.815 for physical frailty; *P* = 0.214 for frailty index). Table 2 shows the associations between frailty and the risks of incident AA, AAA, TAA and AD. After adjusting for a range of potential confounders, including sociodemographic and environmental factors (age, sex, ethnicity, educational level, employment status, family income and TDI), lifestyle (alcohol and tobacco consumption, healthy diet score, physical activity and BMI), and medical condition (SBP, HbA1c, lipid profile and the use of antihypertensive, antidiabetic, and lipid-lowering medications), AA risk was found to be higher in participants with prefrailty (physical frailty: HR 1.24, 95% CI 1.16–1.33; frailty index: HR = 1.67, 95% CI 1.55–1.80) and frailty (physical frailty: HR 1.50, 95% CI 1.28–1.76; frailty index: HR 2.22, 95% CI 1.96–2.51) than in those with non-frailty. The HRs of AA were 1.17 (95% CI 1.13–1.22) and 1.55 (95% CI 1.47–1.64) per one predefined unit increase in physical frailty and the frailty index. Specifically, compared with non-frail participants, participants with frailty had a higher risk of AAA (physical frailty: HR = 1.70, 95% CI 1.41–2.06; frailty index: HR 2.64, 95% CI 2.27–3.06). In contrast, TAA risk exhibited differential associations according to the frailty measure used. The frailty index showed consistent positive associations with TAA risk across categorical analyses (prefrailty: HR 1.47, 95% CI 1.29–1.67; frailty: HR 1.61, 95% CI 1.25–2.07). In comparison, physical frailty showed a weaker and less consistent pattern: prefrailty was modestly associated with higher TAA risk (HR 1.22, 95% CI 1.07–1.38), whereas frailty was not significantly associated with TAA risk (HR 0.99, 95% CI 0.69–1.44). Additionally, for each 1-unit increase in physical frailty, the HRs of AAA and TAA were 1.23 (95% CI 1.17–1.29) and 1.10 (95% CI 1.02–1.18), respectively. Meanwhile, the HRs per 0.1-unit increase in the frailty index were 1.69 (95% CI 1.58–1.81) and 1.35 (95% CI 1.22–1.50) for AAA and TAA, respectively.

**Table 2:** Association of frailty status with incident AA and AD

			Model 1^†^		Model 2^‡^	
	Events	Incidence rate*	HR (95% CI)	*P* value	HR (95% CI)	*P* value
AA						
Physical frailty						
Non-frail	1620	0.427	1.00 (Ref)		1.00 (Ref)	
Prefrail	1851	0.589	1.49 (1.39–1.59)	< 0.001	1.24 (1.16–1.33)	< 0.001
Frail	204	0.872	2.39 (2.07–2.76)	< 0.001	1.50 (1.28–1.76)	< 0.001
Continuous (per 1 unit)	3675	0.512	1.34 (1.30–1.39)	< 0.001	1.17 (1.13–1.22)	< 0.001
Frailty index						
Non-frail	1593	0.366	1.00 (Ref)		1.00 (Ref)	
Prefrail	1681	0.695	1.99 (1.85–2.13)	< 0.001	1.67 (1.55–1.80)	< 0.001
Frail	401	1.002	2.99(2.68–3.34)	< 0.001	2.22 (1.96–2.51)	< 0.001
Continuous (per 0.1 unit)	3675	0.512	1.81 (1.72–1.90)	< 0.001	1.55 (1.47–1.64)	< 0.001
AAA						
Physical frailty						
Non-frail	945	0.249	1.00 (Ref)		1.00 (Ref)	
Prefrail	1190	0.378	1.66 (1.52–1.80)	< 0.001	1.31 (1.20–1.44)	< 0.001
Frail	154	0.658	3.16 (2.67–3.75)	< 0.001	1.70 (1.41–2.06)	< 0.001
Continuous (per 1 unit)	2289	0.319	1.47 (1.40–1.53)	< 0.001	1.23 (1.17–1.29)	< 0.001
Frailty index						
Non-frail	881	0.202	1.00 (Ref)		1.00 (Ref)	
Prefrail	1112	0.460	2.39 (2.19–2.61)	< 0.001	1.86 (1.70–2.05)	< 0.001
Frail	296	0.740	4.04 (3.54–4.61)	< 0.001	2.64 (2.27–3.06)	< 0.001
Continuous (per 0.1 unit)	2289	0.319	2.10 (1.98–2.23)	< 0.001	1.69 (1.58–1.81)	< 0.001
TAA						
Physical frailty						
Non-frail	529	0.139	1.00 (Ref)		1.00 (Ref)	
Prefrail	548	0.174	1.32 (1.17–1.48)	< 0.001	1.22 (1.07–1.38)	0.003
Frail	37	0.158	1.26 (0.90–1.76)	0.171	0.99 (0.69–1.44)	0.980
Continuous (per 1 unit)	1114	0.155	1.16 (1.08–1.24)	< 0.001	1.10 (1.02–1.18)	0.019
Frailty index						
Non-frail	563	0.129	1.00 (Ref)		1.00 (Ref)	
Prefrail	469	0.194	1.55 (1.37–1.75)	< 0.001	1.47 (1.29–1.67)	< 0.001
Frail	82	0.205	1.69 (1.34–2.13)	< 0.001	1.61 (1.25–2.07)	< 0.001
Continuous (per 0.1 unit)	1114	0.155	1.40 (1.28–1.53)	< 0.001	1.35 (1.22–1.50)	< 0.001
AD						
Physical frailty						
Non-frail	158	0.042	1.00 (Ref)		1.00 (Ref)	
Prefrail	141	0.045	1.11 (0.88–1.39)	0.366	1.03 (0.81–1.31)	0.836
Frail	11	0.047	1.21 (0.65–2.23)	0.546	0.71 (0.34–1.49)	0.373
Continuous (per 1 unit)	310	0.043	1.12 (0.98–1.28)	0.096	1.01 (0.87–1.17)	0.912
Frailty index						
Non-frail	164	0.038	1.00 (Ref)		1.00 (Ref)	
Prefrail	122	0.050	1.37 (1.08–1.73)	0.009	1.21 (0.94–1.56)	0.132
Frail	24	0.060	1.66 (1.08–2.55)	0.021	1.26 (0.77–2.05)	0.362
Continuous (per 0.1 unit)	310	0.043	1.32 (1.11–1.57)	0.010	1.16 (0.95–1.42)	0.134

*Abbreviations*: *HR* hazard ratio, *AA* aortic aneurysm, *AAA* abdominal aortic aneurysm, *TAA* thoracic aortic aneurysm

Levels of significance: *P* < 0.05 (Cox regression model)

*The incidence rate was calculated at the end of follow-up of 15.1 years and reported as per 1,000 person-years

†Model 1 was unadjusted

‡Model 2 was adjusted for age, sex, ethnicity, educational level, employment status, family income, TDI, alcohol and tobacco consumption, healthy diet score, physical activity, BMI, SBP, HbA1c, lipid profile and the use of anti-hypertensive medication, anti-hyperglycaemic medication, and lipid-lowering medications

In contrast to the findings for AA and its subtypes, neither physical frailty nor the frailty index showed statistically significant associations with the risk of AD (Table 2). When analyzed as categorical variables, neither prefrailty nor frailty (defined by physical frailty or frailty index) was associated with AD risk (all HRs included 1.00 in their 95% CIs). Similarly, continuous measures of physical frailty (per 1-unit increase: HR 1.01, 95% CI 0.87–1.17, *P* = 0.912) and frailty index (per 0.1-unit increase: HR 1.16, 95% CI 0.95–1.42, *P* = 0.134) did not reach predefined significance thresholds (*P* < 0.05) after full adjustment for covariates.

**Fig. 2:**
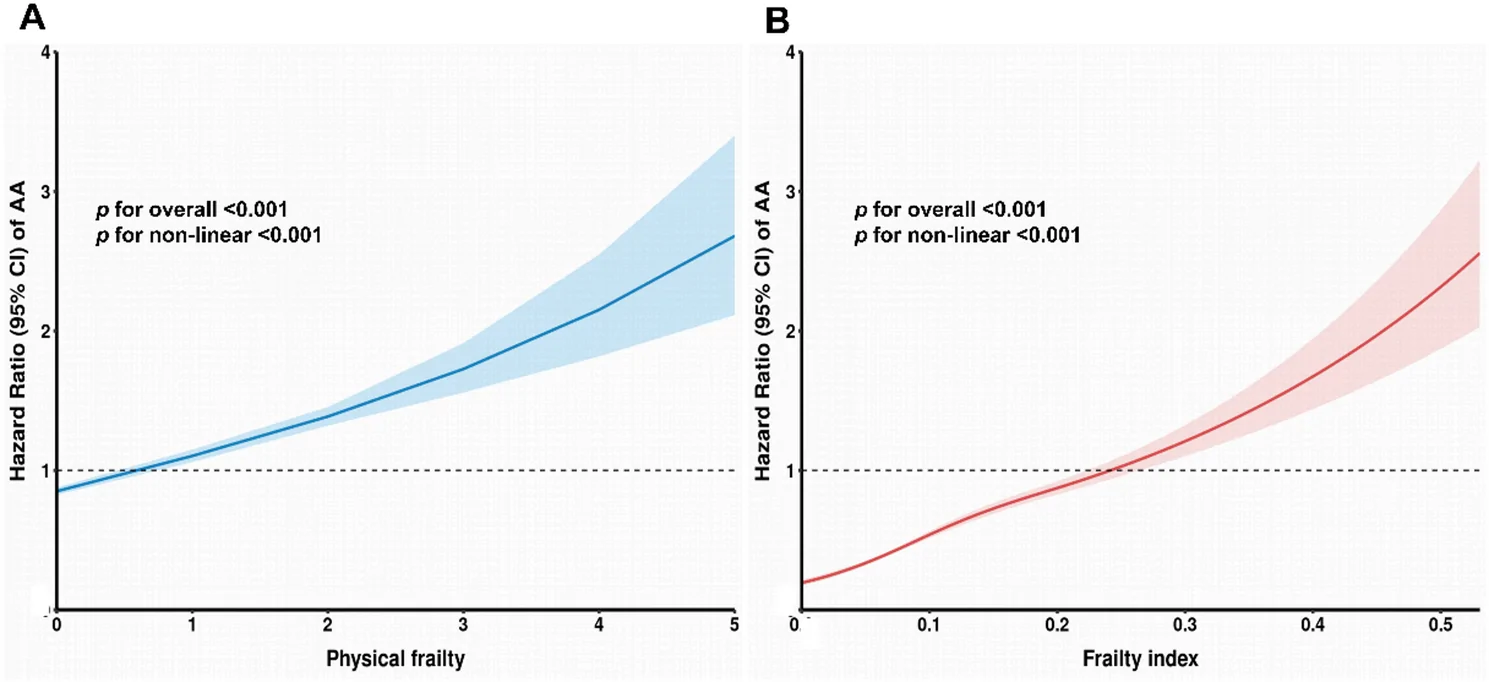
Dose-response association of frailty with the risk of incident AA. (A) Dose-response association between physical frailty and the risk of incident AA. (B) Dose-response association between frailty index and the risk of incident AA. Dark lines represent hazard ratios, and shaded areas represent 95% CIs. Data were fitted using restricted cubic splines with three knots. Models were adjusted for age, sex, ethnicity, educational level, employment status, family income, Townsend deprivation index, alcohol and tobacco consumption, healthy diet score, physical activity, body mass index, systolic blood pressure, glycated haemoglobin, lipid profile, and the use of antihypertensive, antidiabetic, and lipid-lowering medications. Levels of significance: *P* < 0.05. Abbreviations: AA, aortic aneurysm

As shown in Fig. 2, J-shaped associations were found among physical frailty, frailty index, and incident AA risk (*P* for nonlinearity < 0.001), with the risk of AA increasing monotonically across the entire range of frailty scores. Similar J-shaped associations were also observed for the risks of incident AAA (*P* for nonlinearity < 0.001, Figure S2). When analyzing TAA risk, distinct nonlinear patterns emerged between the two frailty metrics (both *P* for overall < 0.001; *P* for nonlinearity < 0.001, Figure S2). Physical frailty showed a triphasic response: initial steep increase, followed by a plateau, and a subtle decline at extreme scores. Frailty index exhibited a biphasic trend: rapid risk escalation transitioning to a sustained plateau. In addition, RCS analyses revealed no statistically or clinically meaningful associations between frailty metrics and AD risk. Physical frailty showed neither overall significance (*P* for overall = 0.349) nor nonlinear trends (*P* for nonlinearity = 0.540), with hazard ratios consistently near unity across all scores. While the frailty index demonstrated nominally significant overall (*P* for overall < 0.001) and nonlinear (*P* for nonlinearity < 0.001) associations, all 95% confidence intervals included the null value throughout the score range, indicating no robust risk elevation.

### Stratified analyses

**Fig. 3:**
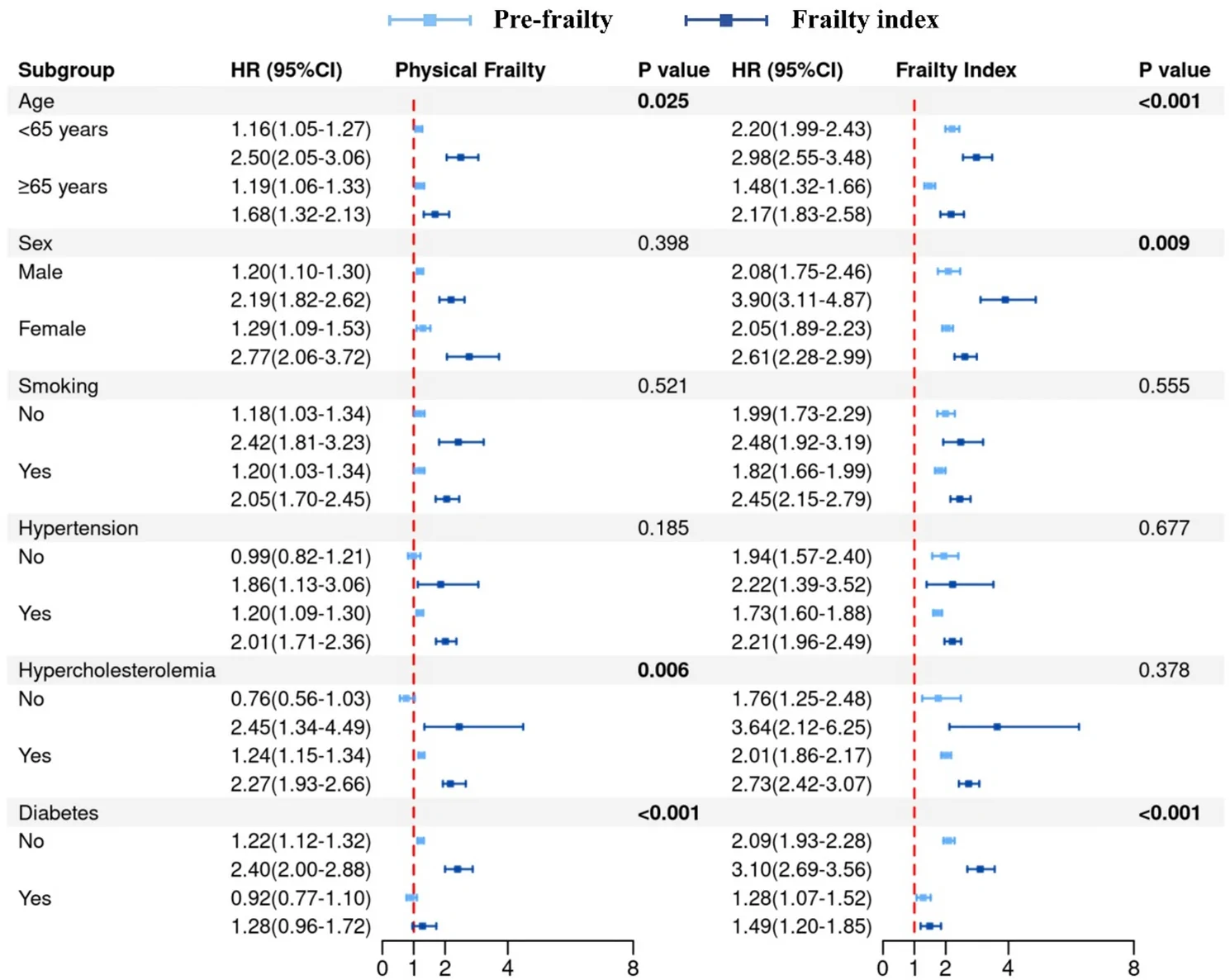
Stratified analysis for the association between frailty status and AA risk. Subgroup analyses were conducted for prefrailty and frailty compared with non-frailty. Multivariable Cox models were adjusted for age, sex, ethnicity, educational level, employment status, family income, Townsend deprivation index, alcohol and tobacco consumption, healthy diet score, physical activity, body mass index, systolic blood pressure, glycated haemoglobin, lipid profile and the use of antihypertensive, antidiabetic, and lipid-lowering medications. Levels of significance: *P* < 0.05. Abbreviations: HR, hazard ratio; AA, aortic aneurysm

The associations of both the frailty index and physical frailty with AA and AAA were more pronounced individuals aged < 65 years (*P* for interaction < 0.05, Fig. 3 and S3) than in other individuals. However, for TAA, a significant age interaction was observed only for the frailty index (*P* for interaction < 0.001) and not for physical frailty (*P* for interaction = 0.111, Figure S4). The association of frailty index with the risk of AA (including AAA and TAA) was more pronounced in male (*P* for interaction = 0.009) than in females, whereas no significant interaction was detected between the physical frailty and sex (*P* for interaction = 0.398; Fig. 3). Hypercholesterolemia specifically modified physical frailty associations in AA (*P* for interaction = 0.006) and TAA (*P* for interaction = 0.018) but not AAA (*P* for interaction = 0.401). Conversely, significant positive associations of both physical frailty and the frailty index with AA and AAA were observed in non-diabetic individuals, and these associations were significantly stronger than those in their diabetic counterparts (*P* for interaction < 0.001 for both frailty measures). All associations converged to null for AD regardless of stratification (Figure S5).

### Sensitivity analysis

The robustness of our primary findings was confirmed through comprehensive sensitivity analyses. Exclusion of participants with baseline cerebral aneurysms, connective tissue diseases, or pre-existing vascular diagnoses (*n* = 404,306) did not materially alter the observed associations between frailty metrics and aortic disease risk (Table S6). Similarly, eliminating cases occurring within the first five years of follow-up (*n* = 473,737) to address potential reverse causation yielded consistent results (Table S7). Multiple imputation of missing covariates produced virtually identical effect estimates (Table S8). Competing risk analyses using Fine-Gray models, accounting for all-cause mortality, demonstrated comparable hazard ratios, confirming the robustness of our findings against competing mortality events (Table S9). These results collectively suggest that our primary analyses were not substantially influenced by pre-existing comorbidities, reverse causation, missing data, or competing mortality risks.

### Propensity score matching

The association between frailty metrics and aortic disease risk persisted in propensity score-matched cohorts, confirming the robustness of our primary findings. Both 1:1 and 1:4 matched analyses (Tables S10-S17) demonstrated similar effect sizes aortic disease outcomes as observed in the main analyses (Table 3 and Table S18). Consistent with our primary results, neither physical frailty nor the frailty index showed statistically significant associations with AD risk in the matched cohorts.

**Table 3:** Association of frailty status with incident AA and AD After Propensity Score Matching (1:1)

	HR (95% CI)	*P* value
AA		
Physical frailty		
Non-frail	1.00 (Ref)	
Prefrail	1.14 (1.06–1.22)	< 0.001
Frail	1.38 (1.20–1.60)	< 0.001
Continuous(per 0.1 unit)	1.11 (1.07–1.15)	< 0.001
Frailty index		
Non-frail	1.00 (Ref)	
Prefrail	1.31 (1.22–1.40)	< 0.001
Frail	1.53(1.37–1.70)	< 0.001
Continuous(per 0.1 unit)	1.26 (1.20–1.32)	< 0.001
AAA		
Physical frailty		
Non-frail	1.00 (Ref)	
Prefrail	1.66 (1.52–1.80)	< 0.001
Frail	3.16 (2.67–3.75)	< 0.001
Continuous(per 0.1 unit)	1.47 (1.40–1.53)	< 0.001
Frailty index		
Non-frail	1.00 (Ref)	
Prefrail	2.39 (2.19–2.61)	< 0.001
Frail	4.04 (3.54–4.61)	< 0.001
Continuous(per 0.1 unit)	2.10 (1.98–2.23)	< 0.001
TAA		
Physical frailty		
Non-frail	1.00 (Ref)	
Prefrail	1.14 (1.01–1.28)	0.035
Frail	1.10 (0.79–1.53)	0.591
Continuous(per 0.1 unit)	1.06 (0.98–1.13)	0.126
Frailty index		
Non-frail	1.00 (Ref)	
Prefrail	1.55 (1.37–1.75)	< 0.001
Frail	1.69 (1.34–2.13)	0.006
Continuous(per 0.1 unit)	1.28 (1.16–1.39)	< 0.001
AD		
Physical frailty		
Non-frail	1.00 (Ref)	
Prefrail	0.90 (0.72–1.13)	0.369
Frail	0.70 (0.38–1.30)	0.261
Continuous(per 0.1 unit)	0.93 (0.81–1.06)	0.259
Frailty index		
Non-frail	1.00 (Ref)	
Prefrail	1.10 (0.87–1.39)	0.440
Frail	1.08 (0.71–1.66)	0.717
Continuous(per 0.1 unit)	1.06 (0.89–1.26)	0.481

Cox regression model was adjusted for age, sex, ethnicity, educational level, employment status, family income, TDI, alcohol and tobacco consumption, healthy diet score, physical activity, BMI, SBP, HbA1c, lipid profile and the use of anti-hypertensive medication, anti-hyperglycaemic medication, and lipid-lowering medications

*Abbreviations*: *HR* hazard ratio, *AA* aortic aneurysm, *AAA* abdominal aortic aneurysm, *TAA* thoracic aortic aneurysm

Levels of significance: *P* <0.05 (Cox regression model)

## Discussion

Our study provides important population-based evidence on the association between frailty and incident aortic disease across distinct clinical phenotypes. We found that both physical frailty and frailty index were associated with higher risks of AA and AAA, whereas the association with TAA was more evident for frailty index than for physical frailty, and no robust association was observed for AD. This association was consistent across two different frailty metrics and robust to various sensitivity analyses, underscoring the potential of frailty as a key marker in identifying individuals at high risk for these aortic diseases. Thus, frailty assessment may provide additional information for identifying individuals with greater vulnerability to aneurysmal aortic disease and may complement established risk factors in risk stratification, particularly for AAA.

Previous research has extensively documented that frailty is both prevalent and associated with poor outcomes in patients with aortic diseases. A pivotal retrospective study involving 5,806 patients undergoing AAA repair found that 46% of the cohort were frail or very frail, with frailty strongly predicting worse surgical outcomes and elevated one-year mortality [25]. Similarly, a large national analysis found that frailty affected 23.4% of patients undergoing thoracic aortic endovascular repair and also remained a strong independent predictor of adverse outcomes [26]. Moreover, frailty has been shown to significantly impact long-term survival in patients with AD, with frail individuals exhibiting considerably lower five-year survival rates despite similar short-term outcomes [27]. Collectively, these findings highlight the relevance of frailty to perioperative risk assessment and postoperative outcomes after aortic repair. Our study extends this body of literature by demonstrating a longitudinal relationship between frailty and an elevated risk of developing aortic diseases.

The mechanisms underlying the frailty-AA relationship appear multifactorial, involving several shared pathophysiological pathways, such as vitamin D deficiency, muscle atrophy, insulin resistance, and systemic inflammation. Vitamin D deficiency, a known contributor to frailty, can exacerbate aortic aneurysm formation by impairing vascular protection, modulating inflammatory responses, and disrupting immune function within the vascular wall [28–31]. Similarly, the loss of muscle mass, a hallmark of frailty, further worsens metabolic dysfunction and increases the risk of surgical complications in AA patients [32–34]. Crucially, shared pathways like insulin resistance and chronic inflammation play pivotal roles in promoting AA development by initiating vascular inflammation, oxidative stress, endothelial dysfunction, and extracellular matrix degradation [35–38]. In addition to degenerative mechanisms, inflammatory and infectious processes may also contribute to selected aneurysm phenotypes, including infective or mycotic aneurysm [39]. Overall, these interconnected pathophysiological mechanisms may help explain the association between frailty and AA, while further studies are needed to determine whether modifying these pathways can reduce aneurysm-related risk.

The different patterns observed for AAA, TAA, and AD may also reflect differences in disease biology and in the scope of the frailty measures used. Physical frailty primarily captures deficits in mobility, strength, energy, weight loss, and physical activity, whereas the frailty index reflects a broader deficit-accumulation construct that includes comorbidities, functional impairments, and psychological or cognitive domains [18, 40]. This broader construct may be more sensitive to multisystem vulnerability, which could explain why the frailty index showed stronger associations with AAA and more consistent associations with TAA than physical frailty alone. Compared with AAA, TAA may be more strongly influenced by medial degeneration, heritable or connective-tissue-related susceptibility, and hemodynamic stress. AD differs further from aneurysmal disease because it often reflects acute structural failure of the aortic wall, frequently in the setting of hypertension, genetic susceptibility, or abrupt mechanical stress [41, 42]. Therefore, the lack of a robust association with AD in our study may reflect differences between chronic aneurysmal degeneration and acute dissection events, rather than a lack of clinical relevance of frailty after AD occurs.

Our study also reveals that frailty’s association with AA risk is particularly pronounced among younger individuals (under 65 years) and non-diabetic individuals. The stronger association among younger participants may reflect accelerated biological aging, with frailty indicating a greater burden of age-related physiological decline than expected for chronological age [43]. In younger individuals, frailty may reflect early vascular aging, heightened inflammatory burden, or other underlying vulnerabilities associated with aortic disease susceptibility. By contrast, among older adults, frailty is often multifactorial and more prevalent, which may dilute its specific contribution to aortic aneurysm development [44]. The association of frailty with aneurysm risk in non-diabetic individuals further highlights the need for nuanced risk assessments. Diabetes has been paradoxically associated with a protective effect on aortic dilation, potentially due to altered extracellular matrix remodeling and aortic wall fibrosis, which may mitigate the effects of frailty in diabetic patients [45]. This protective mechanism may obscure the incremental risk posed by frailty in diabetic individuals, making frailty a more prominent risk factor in non-diabetic populations, where it may exacerbate aortic degeneration through chronic inflammation, oxidative stress, and impaired vascular repair [37, 45, 46]. These subgroup findings suggest that frailty assessment may provide additional risk-stratification information beyond chronological age and diabetes status, although external validation is needed.

Several strengths of this study enhance its validity, including its prospective design, large sample size derived from a well-established cohort, long-term follow-up, and comprehensive adjustment for a wide range of potential confounders. Nevertheless, several limitations warrant careful consideration. First, as an observational study, we cannot infer causality between frailty and aortic aneurysm risk, and residual confounding remains possible despite extensive adjustment and sensitivity analyses. Second, frailty was assessed only at baseline using two validated tools, and changes in frailty status over time were not captured, which may have led to exposure misclassification [47]. Third, some frailty components and covariates were based on self-reported data, introducing the possibility of recall bias and measurement error [11]. Fourth, AA and AD outcomes were identified using linked hospital inpatient records, death registries, and procedure codes. Therefore, undiagnosed asymptomatic aneurysms may have been missed, and we lacked detailed imaging information on aneurysm diameter, morphology, growth rate, and anatomical extent. Fifth, the UK Biobank cohort is predominantly composed of individuals of European ancestry and is subject to healthy volunteer selection, which may limit the generalizability of our findings to other populations or settings with higher disease burdens. Finally, although frailty may provide additional information for individualized risk assessment, the observational nature of this study precludes direct recommendations regarding changes to AAA screening or surveillance practice based on frailty status alone. Further studies are needed to determine whether incorporating frailty into clinical risk models can improve preventive care or follow-up strategies.

## Conclusion

Frailty was associated primarily with incident aneurysmal phenotypes, particularly AA and AAA, whereas the association with TAA was more evident for the frailty index, and no robust association was observed for AD. These associations appeared stronger among younger and non-diabetic participants. Overall, our findings support phenotype-specific associations between frailty and aortic disease and suggest that frailty assessment may provide additional information for aortic risk stratification. Further studies are needed to validate these findings and to determine whether frailty modification can reduce aneurysm-related risk.

## Supplementary Information

Supplementary Material 1.

## Data Availability

The data that support the findings of this study are available from the UK Biobank Resource under established access procedures. This research was conducted using the UK Biobank Resource under Application Number 92668.

## Abbreviations

BMI: Body mass index
TDI: Townsend deprivation index
SBP: Systolic blood pressure
HbA1c: Haemoglobin A1c
