# Supplementary material for: Frailty and prefrailty are associated with increased risk of aortic aneurysm but not aortic dissection: a prospective cohort study of UK biobank participants
Source: BMC Cardiovasc Disord. 2026 Jul 1;26:829. doi: 10.1186/s12872-026-06175-z (PMC13602592; doi:10.1186/s12872-026-06175-z)
Supplement: Supplementary file 1 [file 12872_2026_6175_MOESM1_ESM.docx]

**Supplemental Materials**

**Content:**

**Table S1. Definition of Physical Frailty in the UK Biobank.**

**Table S2. Items from the Baseline UKBB Assessment Used to Compose the Frailty Index.**

**Table S3. Definitions and Sources of Information for AA/AD in the UK Biobank.**

**Table S4.Baseline Characteristics of the Participants in Accordance with Frailty Index.**

**Table S5. Overlap Between Physical Frailty and the Frailty Index.**

**Table S6. Association of Frailty Status with Incident AA /AD after Excluding Individuals Diagnosed with Cerebral Aneurysm, Connective Tissue Disease or Vascular Disease at Baseline.**

**Table S7. Association of Frailty with AA/AD after Excluding Individuals Diagnosed with AA/AD During the First Five Years of Follow-up.**

**Table S8. Association of Frailty with AA/AD Using Multiple Imputation.**

**Table S9. Association of Frailty and AA/AD with Death as a Competing Risk.**

**Table S10. Comparison of Baseline Characteristics Between Groups with and without AA After Propensity Score Matching (1:1)**

**Table S11. Comparison of Baseline Characteristics Between Groups with and without AA After Propensity Score Matching (1:4)**

**Table S12. Comparison of Baseline Characteristics Between Groups with and without AAA After Propensity Score Matching (1:1)**

**Table S13. Comparison of Baseline Characteristics Between Groups with and without AAA After Propensity Score Matching (1:4)**

**Table S14. Comparison of Baseline Characteristics Between Groups with and without TAA After Propensity Score Matching (1:1)**

**Table S15. Comparison of Baseline Characteristics Between Groups with and without TAA After Propensity Score Matching (1:4)**

**Table S16. Comparison of Baseline Characteristics Between Groups with and without AD After Propensity Score Matching (1:1)**

**Table S17. Comparison of Baseline Characteristics Between Groups with and without AD After Propensity Score Matching (1:4)**

**Table S18. Association of frailty status with incident AA and AD After Propensity Score Matching (1:4).**

**Figure S1. Cumulative risks of incident AAA, TAA, AD in accordance with frailty profile.**

**Figure S2. Dose-response association of frailty with the risks of incident AAA, TAA and AD.**

**Figure S3. Stratified analysis for the association between frailty status and AAA risk.**

**Figure S4. Stratified analysis for the association between frailty status and TAA risk.**

**Figure S5. Stratified analysis for the association between frailty status and AD risk.**

**Table S1. Definition of Physical Frailty in the UK Biobank.**

| **Components** | **Self-reported question/**  **measurement in UK**  **Biobank** | **Categories** |
| --- | --- | --- |
| Weight loss | Self-reported: “Compared with one year ago, has your weight changed?” | 1: “Yes, loss weight”;  0: Others  NA: "Do not know/Prefer not to answer" |
| Exhaustion | Self-reported: “Over the past two weeks, how often have you felt tired or had little energy?” | 1: “More than half the days or nearly every day”;  0: Others;  NA: "Do not know/Prefer not to answer" |
| Low physical  Activity | Self-reported: “How many times in the last 4 weeks did you do light DIY?” | 1: “None or light activity with a frequency of once per week or less”;  0: medium or heavy activity, or light activity more than once per week |
| Slow gait speed | Self-reported: “How would you describe your usual walking pace?” | 1: “Slow pace”;  0: Others  NA: "Prefer not to answer" |
| Low grip  strength | Grip strength was measured with a Jamar J00105 hydraulic hand  dynamometer (Lafayette Instrument, Lafayette, IN, USA). Participants were  asked to complete a grip assessment for both hands once. The maximal value of the right and left hands was  used. Measured grip strength expressed in kg by sex- and BMI- adjusted cutoff points. | 1: Male:  ≤29 kg for BMI ≤24 kg/m2 ;  ≤30 kg for BMI 24.1-26 kg/m2 ;  ≤30 kg for BMI 26.1-28 kg/m2 ; or  ≤32 kg for BMI >28 kg/m2 ;  2:Female:  ≤17 kg for BMI ≤23 kg/m2 ;  ≤17.3 kg for BMI 23.1-26 kg/m2 ;  ≤18 kg for BMI 26.1-29 kg/m2 ; or  ≤21 kg for BMI >29 kg/m2 ;  0: Others  NA: data on BMI or grip strength is not available |

**Table S2. Items from the Baseline UKB Assessment Used to Compose the Frailty Index.**

| **Type of deficit** | **Item** | **Trait** | **UKB DataField** | **Coding in Frailty index item** | **Score of 1 n (%)** | **Total** |
| --- | --- | --- | --- | --- | --- | --- |
| Sensory | 1 | Glaucoma | 2227 | Yes = 1  No = 0  Missing if "prefer not to answer" for data-field 6148. | 7543(1.5) | 501729 |
|  |  |  | 6148 |  |  |  |
|  |  |  | 20002 |  |  |  |
|  | 2 | Cataracts | 6148 | Yes = 1  No = 0  Missing if "prefer not to answer"  for data-field 6148. | 16994(3.39) | 501731 |
|  |  |  | 2227 |  |  |  |
|  |  |  | 2002 |  |  |  |
|  | 3 | Hearing difficulty | 2247 | Yes and completely deaf = 1  No = 0 | 122080(25.58) | 477189 |
| Cranial | 4 | Migraine | 2473 | Yes = 1  No = 0  Missing if "prefer not to answer"  for data-field 2473. | 14369(2.87) | 501413 |
|  |  |  | 20002 |  |  |  |
|  | 5 | Dental problems | 6149 | None = 0  Any = 1 | 197501(39.47) | 500358 |
| Mental well being | 6 | Self-rated health | 2178 | Excellent = 0  Good = 0.25  Fair = 0.5  Poor = 1 | 22759(4.56) | 498652 |
|  | 7 | Fatigue: frequency of tiredness / lethargy in last two weeks | 2080 | Not at all = 0  Several days = 0.25  More than half = 0.5  Nearly every day = 1 | 31528(6.50) | 484897 |
|  | 8 | Sleep: experience of  sleeplessness/insomnia | 1200 | Never/rarely = 0  Sometimes = 0.5  Usually = 1 | 141291(28.22) | 500634 |
|  | 9 | Depressed feelings: frequency in last  two weeks | 2050 | Not at all = 0  Several days = 0.5  More than half = 0.75  Nearly every day = 1 | 9500(1.99) | 477982 |
|  | 10 | Self-described nervous personality | 1970 | Yes = 1  No = 0 | 115103(23.62) | 487220 |
|  | 11 | Severe anxiety/ panic attacks | 2473 | Yes = 1  No = 0  Missing if "prefer not to answer"  for data-field 2473. | 6717(1.34) | 501414 |
|  |  |  | 20002 |  |  |  |
|  | 12 | Common to feel loneliness | 2020 | Yes = 1  No = 0 | 91331(18.55) | 492277 |
|  | 13 | Sense of misery (ever/never) | 1930 | Yes = 1  No = 0 | 210449(42.80) | 491751 |
| Infirmity | 14 | Infirmity: long-standing illness or disability | 2188 | Yes = 1  No = 0 | 159788(32.69) | 488787 |
|  | 15 | Falls in last year | 2296 | No falls = 0  Only one fall = 0.5  More than one fall = 1 | 33114(6.63) | 499470 |
|  | 16 | Fractures/broken bones in last five  years | 2463 | Yes = 1  No = 0 | 47434(9.52) | 498335 |
| Cardiometabolic | 17 | Diabetes | 2443 | Yes = 1  No = 0  Missing if "prefer not to answer"  for data-field 2443. | 26544(5.32) | 499586 |
|  |  |  | 20002 |  |  |  |
|  | 18 | Myocardial infarction | 6150 | Yes = 1  No = 0  Missing if "prefer not to answer"  for data-field 6150. | 11760(2.35) | 500853 |
|  |  |  | 20002 |  |  |  |
|  | 19 | Angina | 6150 | Yes = 1  No = 0  Missing if "prefer not to answer"  for data-field 6150. | 16480(3.29) | 500865 |
|  |  |  | 20002 |  |  |  |
|  | 20 | Stroke | 6150 | Yes = 1  No = 0  Missing if "prefer not to answer"  for data-field 6150. | 7807(1.56) | 500852 |
|  |  |  | 20002 |  |  |  |
|  | 21 | High blood pressure | 6150 | Yes = 1  No = 0  Missing if "prefer not to answer"  for data-field 6150. | 137360(27.42) | 500925 |
|  |  |  | 20002 |  |  |  |
|  | 22 | Hypothyroidism | 2473 | Yes = 1  No = 0  Missing if "prefer not to answer"  for data-field 2473. | 24211(4.83) | 501433 |
|  |  |  | 20002 |  |  |  |
|  | 23 | Deep-vein thrombosis | 6152 | Yes = 1  No = 0  Missing if "prefer not to answer"  for data-field 6152. | 9933(1.98) | 500933 |
|  |  |  | 20002 |  |  |  |
|  | 24 | High cholesterol | 6153 | Yes = 1  No = 0  Missing if "prefer not to answer"  for data-field 6177. | 86854(17.31) | 501819 |
|  |  |  | 6177 |  |  |  |
| Respiratory | 25 | Breathing: wheeze in last year | 2316 | Yes = 1  No = 0 | 103211(21.05) | 490325 |
|  | 26 | Pneumonia | 2473 | Yes = 1  No = 0  Missing if "prefer not to answer"  for data-field 2473. | 6899(1.38) | 501406 |
|  |  |  | 20002 |  |  |  |
|  | 27 | Chronic bronchitis/emphysema | 6152 | Yes = 1  No = 0  Missing if "prefer not to answer"  for data-field 6152. | 11248(2.25) | 500946 |
|  |  |  | 20002 |  |  |  |
|  | 28 | Asthma | 6152 | Yes = 1  No = 0  Missing if "prefer not to answer"  for data-field 6152. | 59195(11.82) | 500968 |
|  |  |  | 20002 |  |  |  |
| Musculoskeletal | 29 | Rheumatoid arthritis | 2473 | Yes = 1  No = 0  Missing if "prefer not to answer"  for data-field 2473. | 5656(1.13) | 501410 |
|  |  |  | 20002 |  |  |  |
|  | 30 | Osteoarthritis | 2473 | Yes = 1  No = 0  Missing if "prefer not to answer"  for data-field 2473. | 40662(8.11) | 501473 |
|  |  |  | 20002 |  |  |  |
|  | 31 | Gout | 2473 | Yes = 1  No = 0  Missing if "prefer not to answer"  for data-field 2473. | 6976(1.39) | 501402 |
|  |  |  | 20002 |  |  |  |
|  | 32 | Osteoporosis | 2473 | Yes = 1  No = 0  Missing if "prefer not to answer"  for data-field 2473. | 8034(1.60) | 501415 |
|  |  |  | 20002 |  |  |  |
| Immunological | 33 | Hayfever, allergic rhinitis or eczema | 6152 | Yes = 1  No = 0  Missing if "prefer not to answer"  for data-field 6152. | 121905(24.33) | 500969 |
|  |  |  | 20002 |  |  |  |
|  | 34 | Psoriasis | 2473 | Yes = 1  No = 0  Missing if "prefer not to answer"  for data-field 2473. | 5570(1.11) | 501401 |
|  |  |  | 20002 |  |  |  |
| Cancer | 35 | Any cancer diagnosis | 2453 | Yes = 1  No = 0  Missing if "prefer not to answer"  for data-field 2453. | 44013(8.80) | 500418 |
|  |  |  | 134 |  |  |  |
|  | 36 | Multiple cancers diagnosed (number  reported) | 134 | Range from 0 to 6;  No cancer or single cancer = 0  Multiple cancers = 1 | 2775(0.55) | 501275 |
| Pain | 37 | Chest pain | 2335 | Yes = 1  No = 0 | 80497(16.25) | 495325 |
|  | 38 | Head and/or neck pain | 6159 | Yes (combining responses to pain  in head and neck/shoulders) = 1  No = 0 | 177993(35.54) | 500871 |
|  | 39 | Back pain | 6159 | Yes = 1  No = 0 | 129984(25.95) | 500871 |
|  | 40 | Stomach/abdominal pain | 6159 | Yes = 1  No = 0 | 43965(8.78) | 500871 |
|  | 41 | Hip pain | 6159 | Yes = 1  No = 0 | 56257(11.23) | 500871 |
|  | 42 | Knee pain | 6159 | Yes = 1  No = 0 | 108101(21.58) | 500871 |
|  | 43 | Whole-body pain | 6159 | Yes = 1  No = 0 | 8836(1.76) | 500871 |
|  | 44 | Facial pain | 6159 | Yes = 1  No = 0 | 9333(1.86) | 500871 |
|  | 45 | Sciatica | 2473 | Yes = 1  No = 0  Missing if "prefer not to answer"  for data-field 2473. | 4684(0.93) | 501400 |
|  |  |  | 20002 |  |  |  |
| Gastrointestinal | 46 | Gastric reflux | 2473 | Yes = 1  No = 0  Missing if "prefer not to answer"  for data-field 2473. | 20985(4,18) | 501435 |
|  |  |  | 20002 |  |  |  |
|  | 47 | Hiatus hernia | 2473 | Yes = 1  No = 0  Missing if "prefer not to answer"  for data-field 2473. | 11097(2.21) | 501411 |
|  |  |  | 20002 |  |  |  |
|  | 48 | Gall stones | 2473 | Yes = 1  No = 0  Missing if "prefer not to answer"  for data-field 2473. | 8158(1.63) | 501404 |
|  |  |  | 20002 |  |  |  |
|  | 49 | Diverticulitis | 2473 | Yes = 1  No = 0  Missing if "prefer not to answer"  for data-field 2473. | 5397(1.08) | 501404 |
|  |  |  | 20002 |  |  |  |

**Table S3. Definitions and Sources of Information for AA/AD in the UK Biobank.**

| Outcome | ICD-10 diagnosis | OPCS-4 procedure codes |
| --- | --- | --- |
|  |  |  |
| Aortic aneurysm | I71.1 - I71.9 | L18.1-L18.9, L19.1-L19.9, L25.4, L27.1-L27.3, L27.5, L27.6, L27.8, L27.9, L28.1-L28.3, L28.5, L28.6, L28.8,  L28.9, L46.4 |
| Thoracic aortic aneurysm | I71.1, I71.2 | L18.1, L18.2, L19.1, L19.2, L27.3, L28.3 |
| Abdominal aortic aneurysm | I71.3, I71.4 | L18.3, L18.4, L18.5, L19.3, L19.4, L19.5, L27.1, L27.2, L28.1, L28.2, L46.4 |
| Aortic dissection | I71.0 | L27.4,L28.4 |

**Table S4. Baseline Characteristics of the Participants in Accordance with Frailty Index.**

|  | **Frailty index** | | | | |
| --- | --- | --- | --- | --- | --- |
| **Baseline Characteristics** | **Overall** | **Non-frail** | **Prefrail** | **Frail** | ***P* value** |
| **No.(%)** | 474302 | 287784(60.68) | 160016(33.74) | 26502(5.59) | <0.001 |
| **Follow-up (years)** | 15.12(1.03) | 15.13(0.98) | 15.11(1.10) | 15.10(1.23) | <0.001 |
| **Age (years)** | 56.51(8.08) | 55.10(8.12) | 57.65(7.92) | 58.45(7.48) | <0.001 |
| **Female sex, n (%)** | 258184(54.44) | 152789(53.09) | 89752(56.09) | 15643(59.03) | <0.001 |
| **Ethnicity, n (%)** |  |  |  |  | <0.001 |
| White Europeans | 430487(90.76) | 261734(90.95) | 145047(90.65) | 23706(89.45) |  |
| Non-White Europeans | 42301( 8.92) | 25191(8.75) | 14431(9.02) | 2679(10.11) |  |
| Missing | 1514(0.32) | 859(0.30) | 538(0.34) | 117(0.44) |  |
| **BMI (kg/m2)** | 27.35(4.74) | 26.63(4.22) | 28.21(5.02) | 30.70(6.17) | <0.001 |
| **Employment, n (%)** |  |  |  |  | <0.001 |
| Employment | 254002(53.55) | 172274(59.86) | 74646(46.65) | 7082(26.72) |  |
| Retired | 144337(30.43) | 78386(27.24) | 56188(35.11) | 9763(36.84) |  |
| Unemployment | 71812(15.14) | 34777(12.08) | 27646(17.28) | 9389(35.43) |  |
| Missing | 4151(0.88) | 2347(0.82) | 1536(0.96) | 268(1.01) |  |
| **Education, n (%)** |  |  |  |  | <0.001 |
| Degree-level education | 54658(11.52) | 37662(13.09) | 15350(9.59) | 1646(6.21) |  |
| Non-college education | 336350(70.91) | 211484(73.49) | 109250(68.27) | 15616(58.92) |  |
| Missing | 83294(17.56) | 38638(13.43) | 35416(22.13) | 9240(34.87) |  |
| **Alcohol consumption status, n (%)** |  |  |  |  | <0.001 |
| Never drinkers | 19898(4.20) | 10536(3.66) | 7414(4.63) | 1948(7.35) |  |
| Current or former drinkers | 453974(95.71) | 277069(96.28) | 152404(95.24) | 24501(92.45) |  |
| Missing | 430(0.09) | 179(0.06) | 198(0.12) | 53(0.20) |  |
| **Tobacco consumption status, n (%)** |  |  |  |  | <0.001 |
| Never smokers | 259468(54.71) | 169426(58.87) | 79316(49.57) | 10726(40.47) |  |
| Current or former smokers | 213230(44.96) | 117567(40.85) | 80040(50.02) | 15623(58.95) |  |
| Missing | 1604(0.34) | 791(0.27) | 660(0.41) | 153(0.58) |  |
| **Healthy diet score, n (%)** |  |  |  |  |  |
| 0–1 | 49823(10.50) | 29072(10.10) | 17541(10.96) | 3210(12.11) |  |
| 2–3 | 301306(63.53) | 183622(63.81) | 101227(63.26) | 16457(62.10) |  |
| 4–5 | 123173(25.97) | 70590(26.09) | 41248(25.78) | 6835(25.79) |  |
| **Physical activity, n (%)** |  |  |  |  | <0.001 |
| Never | 30975(6.53) | 18824(6.54) | 10404(6.50) | 1747(6.59) |  |
| Low activity | 17845(3.76) | 10919(3.79) | 5941(3.71) | 985(3.72) |  |
| Medium activity | 371409(78.31) | 225440(78.34) | 125239(78.27) | 20730(78.22) |  |
| High activity | 47339(9.98) | 28559(9.92) | 16147(10.09) | 2633(9.94) |  |
| Missing | 6734(1.42) | 4042(1.40) | 2285(1.43) | 407(1.54) |  |
| **TDI** | -1.35(3.06) | -1.64(2.89) | -1.09(3.17) | 0.21(3.55) | <0.001 |
| **Family income, n (%)** |  |  |  |  | <0.001 |
| <£31 000 | 194099(40.92) | 101451(35.25) | 76284(47.67) | 16364(61.75) |  |
| ≥£31 000 | 213856(45.09) | 149715(52.03) | 59116(36.94) | 5025(18.96) |  |
| Missing | 66347(13.99) | 36618(12.72) | 24616(15.38) | 5113(19.29) |  |
| **SBP (mmHg)** | 137.81(18.61) | 137.30(18.67) | 138.73(18.60) | 138.43(18.47) | <0.001 |
| **HbA1c (%)** | 5.44(0.61) | 5.37(0.48) | 5.54(0.70) | 5.81(0.97) | <0.001 |
| **HDL cholesterol (mmol/L)** | 1.45(0.38) | 1.48(0.38) | 1.41(0.38) | 1.33(0.40) | <0.001 |
| **LDL cholesterol (mmol/L)** | 3.56(0.87) | 3.63(0.84) | 3.48(0.90) | 3.30(0.94) | <0.001 |
| **Triglycerides (mmol/L)** | 1.74(1.02) | 1.66(0.98) | 1.83(1.06) | 2.06(1.19) | <0.001 |
| **Cholesterol (mmol/L)** | 5.70(1.14) | 5.79(1.09) | 5.59(1.19) | 5.34(1.26) | <0.001 |
| **Anti-hypertensive medication, n (%)** | 52687(11.11) | 19925(6.92) | 26436(16.52) | 6326(23.87) | <0.001 |
| **Anti-hyperglycaemic medication, n (%)** | 16247(3.43) | 3151(1.09) | 9341(5.84) | 3755(14.17) | <0.001 |
| **Lipid-lowering medication, n (%)** | 49263(10.39) | 16562(5.76) | 26214(16.38) | 6487(24.48) | <0.001 |

Levels of significance: *P* <0.05 (ANOVA, Kruskal–Wallis H, and chi-squared tests).

Abbreviations: BMI, body mass index; TDI, Townsend deprivation index; SBP, systolic blood pressure; HbA1c, haemoglobin A1c.

**Table S5. Overlap Between Physical Frailty and the Frailty Index.**

| Frailty Index | Physical frailty | | | Total |
| --- | --- | --- | --- | --- |
|  | Non-frail, n(%) | Pre-frail, n(%) | Frailty, n(%) |  |
| Non-frail, n(%) | 176096(37.1%) | 109295(23.0%) | 2393(0..5%) | 287784(60.7%) |
| Pre-frail, n(%) | 68871(14.5%) | 83767(17.7%) | 7378(1.6%) | 160016(33.7%) |
| Frailty, n(%) | 5016(1.1%) | 15711(3.3%) | 5775(1.2%) | 26502(5.6%) |
| Total | 249983(52.7%) | 208773(44.0%) | 15546(3.3%) | 474302 |

Observed agreement:56%; Weighted Kappa: 0.282(95%CI:0.279-0.285)

Spearman correlation: 0.310

**Table S6. Association of Frailty Status with Incident AA/AD after Excluding Individuals Diagnosed with Cerebral Aneurysm, Connective Tissue Disease or Vascular Disease at Baseline.**

|  |  |  | **Model 1**^†^ | |  | **Model 2**^‡^ | |
| --- | --- | --- | --- | --- | --- | --- | --- |
|  | **Events** | **Incidence rate*** | **HR (95% CI)** | ***P* value** |  | **HR (95% CI)** | ***P* value** |
| **AA** |  |  |  |  |  |  |  |
| Physical frailty |  |  |  |  |  |  |  |
| Non-frail | 1143 | 0.470 | 1.00 (Ref) |  |  | 1.00 (Ref) |  |
| Prefrail | 1861 | 0.531 | 1.21 (1.13-1.30) | <0.001 |  | 1.09 (1.01-1.18) | 0.026 |
| Frail | 190 | 0.916 | 2.32 (1.99-2.71) | <0.001 |  | 1.55 (1.31-1.84) | <0.001 |
| Continuous(per 0.1 unit) | 3194 | 0.520 | 1.26 (1.21-1.31) | <0.001 |  | 1.13 (1.08-1.18) | <0.001 |
| Frailty index |  |  |  |  |  |  |  |
| Non-frail | 1098 | 0.397 | 1.00 (Ref) |  |  | 1.00 (Ref) |  |
| Prefrail | 1746 | 0.778 | 2.06 (1.91-2.22) | <0.001 |  | 1.71 (1.57-1.85) | <0.001 |
| Frail | 350 | 1.054 | 2.88 (2.57-3.24) | <0.001 |  | 2.06 (1.81-2.36) | <0.001 |
| Continuous(per 0.1 unit) | 3194 | 0.520 | 1.73 (1.64-1.81) | <0.001 |  | 1.52 (1.43-1.61) | <0.001 |
| **AAA** |  |  |  |  |  |  |  |
| Physical frailty |  |  |  |  |  |  |  |
| Non-frail | 676 | 0.278 | 1.00 (Ref) |  |  | 1.00 (Ref) |  |
| Prefrail | 1184 | 0.338 | 1.32 (1.20-1.45) | <0.001 |  | 1.16 (1.05-1.28) | 0.004 |
| Frail | 134 | 0.646 | 2.84 (2.36-3.42) | <0.001 |  | 1.68 (1.37-2.07) | <0.001 |
| Continuous(per 0.1 unit) | 1994 | 0.324 | 1.34 (1.27-1.41) | <0.001 |  | 1.16 (1.10-1.22) | <0.001 |
| Frailty index |  |  |  |  |  |  |  |
| Non-frail | 737 | 0.226 | 1.00 (Ref) |  |  | 1.00 (Ref) |  |
| Prefrail | 997 | 0.522 | 2.43 (2.21-2.67) | <0.001 |  | 1.87 (1.69-2.07) | <0.001 |
| Frail | 260 | 0.783 | 3.79 (3.31-4.35) | <0.001 |  | 2.41 (2.05-2.82) | <0.001 |
| Continuous(per 0.1 unit) | 1994 | 0.324 | 1.97 (1.85-2.09) | <0.001 |  | 1.63 (1.52-1.75) | <0.001 |
| **TAA** |  |  |  |  |  |  |  |
| Physical frailty |  |  |  |  |  |  |  |
| Non-frail | 364 | 0.150 | 1.00 (Ref) |  |  | 1.00 (Ref) |  |
| Prefrail | 551 | 0.157 | 1.11 (0.97-1.26) | 0.139 |  | 1.04 (0.90-1.19) | 0.510 |
| Frail | 48 | 0.231 | 1.75 (1.29-2.36) | 0.006 |  | 1.50 (1.09-2.07) | 0.14 |
| Continuous(per 0.1 unit) | 963 | 0.157 | 1.14 (1.06-1.23) | <0.001 |  | 1.08 (0.99-1.17) | 0.060 |
| Frailty index |  |  |  |  |  |  |  |
| Non-frail | 296 | 0.135 | 1.00 (Ref) |  |  | 1.00 (Ref) |  |
| Prefrail | 595 | 0.210 | 1.61 (1.40-1.85) | <0.001 |  | 1.51 (1.30-1.76) | <0.001 |
| Frail | 72 | 0.217 | 1.70 (1.40-2.17) | <0.001 |  | 1.56 (1.18-2.05) | <0.001 |
| Continuous(per 0.1 unit) | 963 | 0.157 | 1.37 (1.25-1.51) | <0.001 |  | 1.35 (1.21-1.51) | <0.001 |
| **AD** |  |  |  |  |  |  |  |
| Physical frailty |  |  |  |  |  |  |  |
| Non-frail | 98 | 0.040 | 1.00 (Ref) |  |  | 1.00 (Ref) |  |
| Prefrail | 156 | 0.045 | 1.14 (0.88-1.47) | 0.314 |  | 1.07 (0.82-1.40) | 0.609 |
| Frail | 12 | 0.058 | 1.55 (0.85-2.82) | 0.156 |  | 1.01 (0.50-2.05) | 0.971 |
| Continuous(per 0.1 unit) | 266 | 0.043 | 1.13 (0.88-0.91) | 0.260 |  | 1.05 (0.83-1.33) | 0.682 |
| Frailty index |  |  |  |  |  |  |  |
| Non-frail | 68 | 0.048 | 1.00 (Ref) |  |  | 1.00 (Ref) |  |
| Prefrail | 175 | 0.040 | 1.24 (0.94-1.64) | 0.135 |  | 1.01 (0.75-1.38) | 0.929 |
| Frail | 23 | 0.069 | 1.81 (1.17-2.80) | 0.008 |  | 1.25 (0.74-2.09) | 0.404 |
| Continuous(per 0.1 unit) | 266 | 0.043 | 1.28 (1.06-1.55) | 0.01 |  | 1.07 (0.86-1.35) | 0.531 |

Levels of significance: *p* <0.05 (Cox regression model).

Abbreviations: HR, hazard ratio; AA, aortic aneurysm; AAA, abdominal aortic aneurysm; TAA, thoracic aortic aneurysm.

*The incidence rate was calculated at the end of follow-up of 15.1 years and reported as per 1,000 person-years.

†Model 1 was unadjusted.

‡Model 2 was adjusted forage, sex, ethnicity, educational level, employment status, family income, TDI, alcohol and tobacco consumption, healthy diet score, physical activity, BMI, SBP, HbA1c, lipid profile and the use of anti-hypertensive medication, anti-hyperglycaemic medication, and lipid-lowering medications.

**Table S7. Association of Frailty with AA/AD after Excluding Individuals Diagnosed with AA/AD During the First Five Years of Follow-up.**

|  |  |  | **Model 1**^†^ | | **Model 2**^‡^ | |
| --- | --- | --- | --- | --- | --- | --- |
|  | **Events** | **Incidence rate*** | **HR (95% CI)** | ***P* value** | **HR (95% CI)** | ***P* value** |
| **AA** |  |  |  |  |  |  |
| Physical frailty |  |  |  |  |  |  |
| Non-frail | 1388 | 0.366 | 1.00 (Ref) |  | 1.00 (Ref) |  |
| Prefrail | 1583 | 0.504 | 1.48(1.38-1.60) | <0.001 | 1.25(1.16-1.35) | <0.001 |
| Frail | 167 | 0.714 | 2.28(1.94-2.68) | <0.001 | 1.44(1.21-1.72) | <0.001 |
| Continuous(per 0.1 unit) | 3138 | 0.438 | 1.33(1.28-1.38) | <0.001 | 1.17(1.12-1.22) | <0.001 |
| Frailty index |  |  |  |  |  |  |
| Non-frail | 1378 | 0.317 | 1.00 (Ref) |  | 1.00 (Ref) |  |
| Prefrail | 1426 | 0.590 | 1.95(1.81-2.10) | <0.001 | 1.65(1.53-1.79) | <0.001 |
| Frail | 334 | 0.835 | 2.88(2.56-3.25) | <0.001 | 2.17(1.90-2.48) | <0.001 |
| Continuous(per 0.1 unit) | 3138 | 0.438 | 1.78(1.69-1.87) | <0.001 | 1.53(1.45-1.63) | <0.001 |
| **AAA** |  |  |  |  |  |  |
| Physical frailty |  |  |  |  |  |  |
| Non-frail | 791 | 0.209 | 1.00 (Ref) |  | 1.00 (Ref) |  |
| Prefrail | 993 | 0.316 | 1.65(1.50-1.81) | <0.001 | 1.33(1.21-1.47) | <0.001 |
| Frail | 126 | 0.539 | 3.09(2.56-3.73) | <0.001 | 1.69(1.37-2.09) | <0.001 |
| Continuous(per 0.1 unit) | 1910 | 0.266 | 1.46(1.39-1.53) | <0.001 | 1.23(1.16-1.30) | <0.001 |
| Frailty index |  |  |  |  |  |  |
| Non-frail | 752 | 0.173 | 1.00 (Ref) |  | 1.00 (Ref) |  |
| Prefrail | 914 | 0.378 | 2.30(2.09-2.53) | <0.001 | 1.81(1.64-2.01) | <0.001 |
| Frail | 244 | 0.610 | 3.90(3.38-4.51) | <0.001 | 2.57(2.19-3.03) | <0.001 |
| Continuous(per 0.1 unit) | 1910 | 0.266 | 2.06(1.93-2.19) | <0.001 | 1.66(1.54-1.79) | <0.001 |
| **TAA** |  |  |  |  |  |  |
| Physical frailty |  |  |  |  |  |  |
| Non-frail | 457 | 0.121 | 1.00 (Ref) |  | 1.00 (Ref) |  |
| Prefrail | 482 | 0.153 | 1.34(1.18-1.52) | <0.001 | 1.24(1.08-1.42) | 0.002 |
| Frail | 33 | 0.141 | 1.31(0.92-1.86) | 0.140 | 1.01(0.68-1.50) | 0.959 |
| Continuous(per 0.1 unit) | 972 | 0.136 | 1.17(1.08-1.26) | <0.001 | 1.10(1.02-1.20) | 0.018 |
| Frailty index |  |  |  |  |  |  |
| Non-frail | 486 | 0.112 | 1.00 (Ref) |  | 1.00 (Ref) |  |
| Prefrail | 416 | 0.172 | 1.59(1.40-1.81) | <0.001 | 1.53(1.33-1.76) | <0.001 |
| Frail | 70 | 0.175 | 1.67(1.30-2.14) | <0.001 | 1.64(1.25-2.15) | <0.001 |
| Continuous(per 0.1 unit) | 972 | 0.136 | 1.41(1.28-1.56) | <0.001 | 1.39(1.24-1.55) | <0.001 |
| **AD** |  |  |  |  |  |  |
| Physical frailty |  |  |  |  |  |  |
| Non-frail | 127 | 0.033 | 1.00 (Ref) |  | 1.00 (Ref) |  |
| Prefrail | 116 | 0.037 | 1.14(0.88-1.46) | 0.320 | 1.08(0.83-1.41) | 0.579 |
| Frail | 9 | 0.038 | 1.23(0.62-2.42) | 0.551 | 0.82(0.37-1.80) | 0.614 |
| Continuous(per 0.1 unit) | 252 | 0.035 | 1.12(0.97-1.30) | 0.119 | 1.05(0.89-1.23) | 0.559 |
| Frailty index |  |  |  |  |  |  |
| Non-frail | 138 | 0.032 | 1.00 (Ref) |  | 1.00 (Ref) |  |
| Prefrail | 98 | 0.04 | 1.31(1.01-1.69) | 0.044 | 1.18(0.90-1.56) | 0.235 |
| Frail | 16 | 0.040 | 1.32(0.78-1.69) | 0.300 | 1.08(0.61-1.91) | 0.782 |
| Continuous(per 0.1 unit) | 252 | 0.035 | 1.22(1.01-1.48) | 0.048 | 1.11(0.89-1.38) | 0.356 |

Levels of significance: *P* <0.05 (Cox regression model).

Abbreviations: HR, hazard ratio; AA, aortic aneurysm; AAA, abdominal aortic aneurysm; TAA, thoracic aortic aneurysm.

*The incidence rate was calculated at the end of follow-up of 15.1 years and reported as per 1,000 person-years.

†Model 1 was unadjusted.

‡Model 2 was adjusted forage, sex, ethnicity, educational level, employment status, family income, TDI, alcohol and tobacco consumption, healthy diet score, physical activity, BMI, SBP, HbA1c, lipid profile and the use of anti-hypertensive medication, anti-hyperglycaemic medication, and lipid-lowering medications.

**Table S8. Association of Frailty with AA/AD Using Multiple Imputation.**

|  |  |  | **Model 1**^†^ | | **Model 2**^‡^ | |
| --- | --- | --- | --- | --- | --- | --- |
|  | **Events** | **Incidence rate*** | **HR (95% CI)** | ***P* value** | **HR (95% CI)** | ***P* value** |
| **AA** |  |  |  |  |  |  |
| Physical frailty |  |  |  |  |  |  |
| Non-frail | 1620 | 0.427 | 1.00 (Ref) |  | 1.00 (Ref) |  |
| Prefrail | 1851 | 0.589 | 1.49 (1.39-1.59) | <0.001 | 1.26(1.17-1.35) | <0.001 |
| Frail | 204 | 0.872 | 2.39 (2.07-2.76) | <0.001 | 1.58(1.34-1.85) | <0.001 |
| Continuous(per 0.1 unit) | 3675 | 0.512 | 1.34 (1.30-1.39) | <0.001 | 1.19(1.14-1.24) | <0.001 |
| Frailty index |  |  |  |  |  |  |
| Non-frail | 1593 | 0.366 | 1.00 (Ref) |  | 1.00 (Ref) |  |
| Prefrail | 1681 | 0.695 | 1.99 (1.85-2.13) | <0.001 | 1.70(1.58-1.83) | <0.001 |
| Frail | 401 | 1.002 | 2.99(2.68-3.34) | <0.001 | 2.30(2.04-2.60) | <0.001 |
| Continuous(per 0.1 unit) | 3675 | 0.512 | 1.81 (1.72-1.90) | <0.001 | 1.58(1.49-1.66) | <0.001 |
| **AAA** |  |  |  |  |  |  |
| Physical frailty |  |  |  |  |  |  |
| Non-frail | 945 | 0.249 | 1.00 (Ref) |  | 1.00 (Ref) |  |
| Prefrail | 1190 | 0.378 | 1.66 (1.52-1.80) | <0.001 | 1.35(1.23-1.47) | <0.001 |
| Frail | 154 | 0.658 | 3.16 (2.67-3.75) | <0.001 | 1.83(1.51-2.21) | <0.001 |
| Continuous(per 0.1 unit) | 2289 | 0.319 | 1.47 (1.40-1.53) | <0.001 | 1.25(1.19-1.31) | <0.001 |
| Frailty index |  |  |  |  |  |  |
| Non-frail | 881 | 0.202 | 1.00 (Ref) |  | 1.00 (Ref) |  |
| Prefrail | 1112 | 0.460 | 2.39 (2.19-2.61) | <0.001 | 1.91(1.74-2.10) | <0.001 |
| Frail | 296 | 0.740 | 4.04 (3.54-4.61) | <0.001 | 2.79(2.40-3.23) | <0.001 |
| Continuous(per 0.1 unit) | 2289 | 0.319 | 2.10 (1.98-2.23) | <0.001 | 1.73(1.62-1.85) | <0.001 |
| **TAA** |  |  |  |  |  |  |
| Physical frailty |  |  |  |  |  |  |
| Non-frail | 529 | 0.139 | 1.00 (Ref) |  | 1.00 (Ref) |  |
| Prefrail | 548 | 0.174 | 1.32 (1.17-1.48) | <0.001 | 1.22(1.08-1.39) | 0.002 |
| Frail | 37 | 0.158 | 1.26 (0.90-1.76) | 0.171 | 1.01(0.70-1.46) | 0.970 |
| Continuous(per 0.1 unit) | 1114 | 0.155 | 1.16 (1.08-1.24) | <0.001 | 1.10(1.02-1.19) | 0.015 |
| Frailty index |  |  |  |  |  |  |
| Non-frail | 563 | 0.129 | 1.00 (Ref) |  | 1.00 (Ref) |  |
| Prefrail | 469 | 0.194 | 1.55 (1.37-1.75) | <0.001 | 1.47(1.29-1.68) | <0.001 |
| Frail | 82 | 0.205 | 1.69 (1.34-2.13) | <0.001 | 1.62(1.26-2.09) | <0.001 |
| Continuous(per 0.1 unit) | 1114 | 0.155 | 1.40 (1.28-1.53) | <0.001 | 1.36(1.23-1.50) | <0.001 |
| **AD** |  |  |  |  |  |  |
| Physical frailty |  |  |  |  |  |  |
| Non-frail | 158 | 0.042 | 1.00 (Ref) |  | 1.00 (Ref) |  |
| Prefrail | 141 | 0.045 | 1.11 (0.88-1.39) | 0.366 | 1.03(0.81-1.31) | 0.835 |
| Frail | 11 | 0.047 | 1.21 (0.65-2.23) | 0.546 | 0.72(0.35-1.50) | 0.382 |
| Continuous(per 0.1 unit) | 310 | 0.043 | 1.12 (0.98-1.28) | 0.096 | 1.01(0.87-1.17) | 0.905 |
| Frailty index |  |  |  |  |  |  |
| Non-frail | 164 | 0.038 | 1.00 (Ref) |  | 1.00 (Ref) |  |
| Prefrail | 122 | 0.050 | 1.37 (1.08-1.73) | 0.009 | 1.21(0.94-1.56) | 0.135 |
| Frail | 24 | 0.060 | 1.66 (1.08-2.55) | 0.021 | 1.26(0.77-2.05) | 0.357 |
| Continuous(per 0.1 unit) | 310 | 0.043 | 1.32 (1.11-1.57) | 0.010 | 1.16(0.95-1.41) | 0.134 |

Levels of significance: *P* <0.05 (Cox regression model).

Abbreviations: HR, hazard ratio; AA, aortic aneurysm; AAA, abdominal aortic aneurysm; TAA, thoracic aortic aneurysm.

*The incidence rate was calculated at the end of follow-up of 15.1 years and reported as per 1,000 person-years.

†Model 1 was unadjusted.

‡Model 2 was adjusted forage, sex, ethnicity, educational level, employment status, family income, TDI, alcohol and tobacco consumption, healthy diet score, physical activity, BMI, SBP, HbA1c, lipid profile and the use of anti-hypertensive medication, anti-hyperglycaemic medication, and lipid-lowering medications.

**Table S9. Association of Frailty and AA/AD with Death as a Competing Risk.**

|  |  |  | **Fully-adjusted Model** | |
| --- | --- | --- | --- | --- |
|  | **Events** | **Incidence rate** | **HR (95% CI)** | ***P* value** |
| **AA** |  |  |  |  |
| Physical frailty |  |  |  |  |
| Non-frail | 1620 | 0.427 | 1.00 (Ref) |  |
| Prefrail | 1851 | 0.589 | 1.29(1.20-1.38) | <0.001 |
| Frail | 204 | 0.872 | 1.58(1.35-1.86) | <0.001 |
| Continuous(per 0.1 unit) | 3675 | 0.512 | 1.19(1.15-1.24) | <0.001 |
| Frailty index |  |  |  |  |
| Non-frail | 1593 | 0.366 | 1.00 (Ref) |  |
| Prefrail | 1681 | 0.695 | 1.66(1.54-1.78) | <0.001 |
| Frail | 401 | 1.002 | 2.12(1.88-2.40) | <0.001 |
| Continuous(per 0.1 unit) | 3675 | 0.512 | 1.52(1.44-1.60) | <0.001 |
| **AAA** |  |  |  |  |
| Physical frailty |  |  |  |  |
| Non-frail | 945 | 0.249 | 1.00 (Ref) |  |
| Prefrail | 1190 | 0.378 | 1.31(1.19-1.43) | <0.001 |
| Frail | 154 | 0.658 | 1.77(1.47-2.13) | <0.001 |
| Continuous(per 0.1 unit) | 2289 | 0.319 | 1.23(1.17-1.29) | <0.001 |
| Frailty index |  |  |  |  |
| Non-frail | 881 | 0.202 | 1.00 (Ref) |  |
| Prefrail | 1112 | 0.460 | 1.87(1.70-2.05) | <0.001 |
| Frail | 296 | 0.740 | 2.55(2.20-2.96) | <0.001 |
| Continuous(per 0.1 unit) | 2289 | 0.319 | 1.67(1.56-1.78) | <0.001 |
| **TAA** |  |  |  |  |
| Physical frailty |  |  |  |  |
| Non-frail | 529 | 0.139 | 1.00 (Ref) |  |
| Prefrail | 548 | 0.174 | 1.21(1.07-1.37) | 0.002 |
| Frail | 37 | 0.158 | 1.04(0.73-1.46) | 0.850 |
| Continuous(per 0.1 unit) | 1114 | 0.155 | 1.09(1.01-1.17) | 0.020 |
| Frailty index |  |  |  |  |
| Non-frail | 563 | 0.129 | 1.00 (Ref) |  |
| Prefrail | 469 | 0.194 | 1.43(1.26-1.63) | <0.001 |
| Frail | 82 | 0.205 | 1.47(1.14-1.88) | 0.003 |
| Continuous(per 0.1 unit) | 1114 | 0.155 | 1.31(1.18-1.44) | <0.001 |
| **AD** |  |  |  |  |
| Physical frailty |  |  |  |  |
| Non-frail | 158 | 0.042 | 1.00 (Ref) |  |
| Prefrail | 141 | 0.045 | 0.98(0.77-1.24) | 0.840 |
| Frail | 11 | 0.047 | 0.85(0.44-1.63) | 0.620 |
| Continuous(per 0.1 unit) | 310 | 0.043 | 1.01(0.87-1.17) | 0.901 |
| Frailty index |  |  |  |  |
| Non-frail | 164 | 0.038 | 1.00 (Ref) |  |
| Prefrail | 122 | 0.050 | 1.21(0.94-1.55) | 0.150 |
| Frail | 24 | 0.060 | 1.26(0.79-2.00) | 0.330 |
| Continuous(per 0.1 unit) | 310 | 0.043 | 1.16(0.95-1.41) | 0.140 |

Levels of significance: *P* <0.05 (Cox regression model).

Abbreviations: HR, hazard ratio; AA, aortic aneurysm; AAA, abdominal aortic aneurysm; TAA, thoracic aortic aneurysm.

Fully-adjusted Model was adjusted for age, sex, ethnicity, educational level, employment status, family income, TDI, alcohol and tobacco consumption, healthy diet score, physical activity, BMI, SBP, HbA1c, lipid profile and the use of anti-hypertensive medication, anti-hyperglycaemic medication, and lipid-lowering medications.

**Table S10. Comparison of Baseline Characteristics Between Groups with and without AA After Propensity Score Matching (1:1)**

| **Baseline Characteristics** | **No AA**  **(N = 3675)** | **AA**  **(N = 3675)** | ***P*-value** |
| --- | --- | --- | --- |
| **Age (years)** | 62.11(6.05) | 62.20(5.89) | 0.487 |
| **Female sex, n (%)** | 776(21.12) | 785(21.36) | 0.820 |
| **Ethnicity, n (%)** |  |  | 0.155 |
| White Europeans | 3387(92.16) | 3437(93.52) |  |
| Non-White Europeans | 276(7.51) | 227(6.18) |  |
| Missing | 12(0.33) | 11(0.30) |  |
| **BMI (kg/m2)** | 28.42(4.68) | 28.29(4.42) | 0.215 |
| **Employment, n (%)** |  |  | 0.792 |
| Employment | 1168(31.78) | 1184(32.22) |  |
| Retired | 1884(51.27) | 1897(51.62) |  |
| Unemployment | 588(16.00) | 564(15.35) |  |
| Missing | 35(0.95) | 30(0.82) |  |
| **Education, n (%)** |  |  | 0.636 |
| Degree-level education | 316(8.60) | 336(9.14) |  |
| Non-college education | 2257(61.41) | 2262(61.55) |  |
| Missing | 1102(29.99) | 1077(29.31) |  |
| **Alcohol consumption status, n (%)** |  |  | 0.454 |
| Never drinkers | 101(2.75) | 99(2.69) |  |
| Current or former drinkers | 3564(96.98) | 3568(97.09) |  |
| Missing | 10(0.27) | 8(0.22) |  |
| **Tobacco consumption status, n (%)** |  |  | 0.343 |
| Never smokers | 1106(30.10) | 1117(30.39) |  |
| Current or former smokers | 2540(69.12) | 2539(69.90) |  |
| Missing | 29(0.29) | 19(0.52) |  |
| **Healthy diet score, n (%)** |  |  | 0.854 |
| 0–1 | 482(13.12) | 475(12.93) |  |
| 2–3 | 2330(63.40) | 2317(63.05) |  |
| 4–5 | 863(23.48) | 883(24.03) |  |
| **Physical activity, n (%)** |  |  | 0.690 |
| Never | 213(5.80) | 220(5.99) |  |
| Low activity | 148(4.03) | 148(4.03) |  |
| Medium activity | 2909(79.20) | 282(78.40) |  |
| High activity | 354(9.63) | 383(10.40) |  |
| Missing | 51(1.39) | 42(1.14) |  |
| **TDI** | -1.22(3.16) | -1.20(3.11) | 0.746 |
| **Family income, n (%)** |  |  | 0.274 |
| <£31 000 | 2032(55.29) | 2003(54.50) |  |
| ≥£31 000 | 1173(31.92) | 1155(31.43) |  |
| Missing | 420(12.79) | 517(14.07) |  |
| **SBP (mmHg)** | 142.29(18.16) | 142.98(18.75) | 0.490 |
| **HbA1c (%)** | 5.58(0.63) | 5.57(0.60) | 0.635 |
| **HDL cholesterol (mmol/L)** | 1.29(0.33) | 1.29(0.35) | 0.793 |
| **LDL cholesterol (mmol/L)** | 3.43(0.90) | 3.44(0.91) | 0.477 |
| **Triglycerides (mmol/L)** | 2.02(1.12) | 2.00(1.13) | 0.517 |
| **Cholesterol (mmol/L)** | 5.44(1.21) | 5.45(1.19) | 0.953 |
| **Anti-hypertensive medication, n (%)** | 1288(35.05) | 1282(34.88) | 0.723 |
| **Anti-hyperglycaemic medication, n (%)** | 130(3.54) | 132(3.59) | 0.575 |
| **Lipid-lowering medication, n (%)** | 1223(33.28) | 1216(33.09) | 0.647 |
| **Physical frailty, n (%)** |  |  |  |
| Non-frail | 1772(48.22) | 1620(44.08) | <0.001 |
| Prefrail | 1753(47.70) | 1851(50.37) |  |
| Frail | 150(4.08) | 204(5.55) |  |
| Continuous(per 0.1 unit) | 0.75(0.88) | 0.82(0.90) | <0.001 |
| **Frailty index, n (%)** |  |  |  |
| Non-frail | 1959(53.31) | 1593(43.35) | <0.001 |
| Prefrail | 1436(39.07) | 1681(45.74) |  |
| Frail | 280(7.62) | 401(10.91) |  |
| Continuous(per 0.1 unit) | 1.56(0.64) | 1.68(0.66) | <0.001 |

Levels of significance: *P* <0.05 (ANOVA, Kruskal–Wallis H, and chi-squared tests).

Abbreviations: BMI, body mass index; TDI, Townsend deprivation index; SBP, systolic blood pressure; HbA1c, haemoglobin A1c.

**Table S11. Comparison of Baseline Characteristics Between Groups with and without AA After Propensity Score Matching (1:4)**

| **Baseline Characteristics** | **No AA**  **(N = 14700)** | **AA**  **(N = 3675)** | ***P*-value** |
| --- | --- | --- | --- |
| **Age (years)** | 62.23(6.00) | 62.20(5.89) | 0.996 |
| **Female sex, n (%)** | 3137(21.34) | 785(21.36) | 0.820 |
| **Ethnicity, n (%)** |  |  | 0.563 |
| White Europeans | 13754(93.56) | 3437(93.52) |  |
| Non-White Europeans | 899(6.12) | 227(6.18) |  |
| Missing | 47(0.32) | 11(0.30) |  |
| **BMI (kg/m2)** | 28.28(4.42) | 28.29(4.42) | 0.943 |
| **Employment, n (%)** |  |  | 0.969 |
| Employment | 4706(32.10) | 1184(32.22) |  |
| Retired | 7614(51.80) | 1897(51.62) |  |
| Unemployment | 2270(15.44) | 564(15.35) |  |
| Missing | 110(0.75) | 30(0.82) |  |
| **Education, n (%)** |  |  | 0.879 |
| Degree-level education | 1384(9.41) | 336(9.14) |  |
| Non-college education | 9025(61.39) | 2262(61.55) |  |
| Missing | 4291(29.19) | 1077(29.31) |  |
| **Alcohol consumption status, n (%)** |  |  | 0.344 |
| Never drinkers | 375(2.55) | 99(2.69) |  |
| Current or former drinkers | 14307(97.33) | 3568(97.09) |  |
| Missing | 18(0.12) | 8(0.22) |  |
| **Tobacco consumption status, n (%)** |  |  | 0.215 |
| Never smokers | 4532(30.83) | 1117(30.39) |  |
| Current or former smokers | 10089(68.63) | 2539(69.90) |  |
| Missing | 79(0.54) | 19(0.52) |  |
| **Healthy diet score, n (%)** |  |  | 0.909 |
| 0–1 | 1937(13.18) | 475(12.93) |  |
| 2–3 | 9260(62.99) | 2317(63.05) |  |
| 4–5 | 3503(23.83) | 883(24.03) |  |
| **Physical activity, n (%)** |  |  | 0.969 |
| Never | 918(6.24) | 220(5.99) |  |
| Low activity | 606(4.12) | 148(4.03) |  |
| Medium activity | 11494(78.19) | 282(78.40) |  |
| High activity | 1507(10.25) | 383(10.40) |  |
| Missing | 175(1.19) | 42(1.14) |  |
| **TDI** | -1.20(3.15) | -1.20(3.11) | 0.914 |
| **Family income, n (%)** |  |  | 0.863 |
| <£31 000 | 8077(54.95) | 2003(54.50) |  |
| ≥£31 000 | 4596(31.27) | 1155(31.43) |  |
| Missing | 2027(13.79) | 517(14.07) |  |
| **SBP (mmHg)** | 142.93(18.46) | 142.98(18.75) | 0.883 |
| **HbA1c (%)** | 5.58(0.62) | 5.57(0.60) | 0.804 |
| **HDL cholesterol (mmol/L)** | 1.29(0.33) | 1.29(0.35) | 0.774 |
| **LDL cholesterol (mmol/L)** | 3.45(0.92) | 3.44(0.91) | 0.770 |
| **Triglycerides (mmol/L)** | 2.00(1.10) | 2.00(1.13) | 0.856 |
| **Cholesterol (mmol/L)** | 5.46(1.22) | 5.45(1.19) | 0.665 |
| **Anti-hypertensive medication, n (%)** | 5078(34.54) | 1282(34.88) | 0.713 |
| **Anti-hyperglycaemic medication, n (%)** | 522(3.55) | 132(3.59) | 0.575 |
| **Lipid-lowering medication, n (%)** | 4813(32.74) | 1216(33.09) | 0.703 |
| **Physical frailty, n (%)** |  |  |  |
| Non-frail | 6958(47.33) | 1620(44.08) | <0.001 |
| Prefrail | 7075(48.13) | 1851(50.37) |  |
| Frail | 667(4.54) | 204(5.55) |  |
| Continuous(per 0.1 unit) | 0.75(0.86) | 0.82(0.90) | <0.001 |
| **Frailty index, n (%)** |  |  |  |
| Non-frail | 7468(50.80) | 1593(43.35) | <0.001 |
| Prefrail | 5999(40.81) | 1681(45.74) |  |
| Frail | 1233(8.39) | 401(10.91) |  |
| Continuous(per 0.1 unit) | 1.58(0.64) | 1.68(0.66) | <0.001 |

Levels of significance: *P* <0.05 (ANOVA, Kruskal–Wallis H, and chi-squared tests).

Abbreviations: BMI, body mass index; TDI, Townsend deprivation index; SBP, systolic blood pressure; HbA1c, haemoglobin A1c.

**Table S12. Comparison of Baseline Characteristics Between Groups with and without AAA After Propensity Score Matching (1:1)**

| **Baseline Characteristics** | **No AAA**  **(N = 2289)** | **AAA**  **(N =2289)** | ***P*-value** |
| --- | --- | --- | --- |
| **Age (years)** | 63.31(5.21) | 63.36(4.96) | 0.721 |
| **Female sex, n (%)** | 378(16.51) | 382(16.69) | 0.905 |
| **Ethnicity, n (%)** |  |  | 0.113 |
| White Europeans | 2117(92.49) | 2156(94.19) |  |
| Non-White Europeans | 160(6.99) | 125(5.46) |  |
| Missing | 12(0.52) | 8(0.35) |  |
| **BMI (kg/m2)** | 28.43(4.11) | 28.49(4.37) | 0.629 |
| **Employment, n (%)** |  |  | 0.848 |
| Employment | 613(26.78) | 627(27.39) |  |
| Retired | 1307(57.10) | 1281(55.96) |  |
| Unemployment | 354(15.47) | 363(15.86) |  |
| Missing | 15(0.66) | 18(0.79) |  |
| **Education, n (%)** |  |  | 0.828 |
| Degree-level education | 152(6.64) | 159(6.59) |  |
| Non-college education | 1339(58.50) | 1349(58.93) |  |
| Missing | 798(34.86) | 781(34.12) |  |
| **Alcohol consumption status, n (%)** |  |  | 0.205 |
| Never drinkers | 47(2.05) | 53(2.32) |  |
| Current or former drinkers | 2240(97.86) | 2229(97.38) |  |
| Missing | 2(0.09) | 7(0.31) |  |
| **Tobacco consumption status, n (%)** |  |  | 0.725 |
| Never smokers | 451(19.70) | 446(19.48) |  |
| Current or former smokers | 1820(79.51) | 1829(79.90) |  |
| Missing | 18(0.79) | 14(0.61) |  |
| **Healthy diet score, n (%)** |  |  | 0.977 |
| 0–1 | 322(14.07) | 317(13.85) |  |
| 2–3 | 1430(62.47) | 1433(62.20) |  |
| 4–5 | 537(23.46) | 539(23.55) |  |
| **Physical activity, n (%)** |  |  | 0.859 |
| Never | 125(5.46) | 133(5.81) |  |
| Low activity | 100(4.37) | 91(3.98) |  |
| Medium activity | 1793(78.33) | 1802(78.72) |  |
| High activity | 248(10.83) | 236(10.31) |  |
| Missing | 23(1.04) | 27(1.18) |  |
| **TDI** | -1.05(3.23) | -1.10(3.14) | 0.554 |
| **Family income, n (%)** |  |  | 0.757 |
| <£31 000 | 1343(58.67) | 1360(59.41) |  |
| ≥£31 000 | 576(25.16) | 577(25.21) |  |
| Missing | 370(16.16) | 32(15.38) |  |
| **SBP (mmHg)** | 144.50(18.20) | 144.29(18.70) | 0.703 |
| **HbA1c (%)** | 5.57(0.61) | 5.58(0.62) | 0.439 |
| **HDL cholesterol (mmol/L)** | 1.24(0.31) | 1.23(0.32) | 0.513 |
| **LDL cholesterol (mmol/L)** | 3.43(0.96) | 3.42(0.95) | 0.805 |
| **Triglycerides (mmol/L)** | 2.06(1.11) | 2.10(1.17) | 0.258 |
| **Cholesterol (mmol/L)** | 5.39(1.28) | 5.39(1.25) | 0.877 |
| **Anti-hypertensive medication, n (%)** | 890(38.88) | 895(39.10) | 0.904 |
| **Anti-hyperglycaemic medication, n (%)** | 90(3.93) | 87(3.80) | 0.879 |
| **Lipid-lowering medication, n (%)** | 929(40.59) | 923(40.32) | 0.880 |
| **Physical frailty, n (%)** |  |  |  |
| Non-frail | 1050(45.87) | 945(41.28) | 0.003 |
| Prefrail | 1117(48.80) | 1190(51.99) |  |
| Frail | 122(5.33) | 154(6.73) |  |
| Continuous(per 0.1 unit) | 0.78(0.89) | 0.89(0.94) | <0.001 |
| **Frailty index, n (%)** |  |  |  |
| Non-frail | 1055(46.09) | 881(38.49) | <0.001 |
| Prefrail | 1011(44.17) | 1112(48.58) |  |
| Frail | 223(9.74) | 296(12.93) |  |
| Continuous(per 0.1 unit) | 1.64(0.65) | 1.74(0.67) | <0.001 |

Levels of significance: *P* <0.05 (ANOVA, Kruskal–Wallis H, and chi-squared tests).

Abbreviations: BMI, body mass index; TDI, Townsend deprivation index; SBP, systolic blood pressure; HbA1c, haemoglobin A1c.

**Table S13. Comparison of Baseline Characteristics Between Groups with and without AAA After Propensity Score Matching (1:4)**

| **Baseline Characteristics** | **No AAA**  **(N = 9156)** | **AAA**  **(N = 2289)** | ***P*-value** |
| --- | --- | --- | --- |
| **Age (years)** | 63.42(5.09) | 63.36(4.96) | 0.633 |
| **Female sex, n (%)** | 1541(16.83) | 382(16.69) | 0.896 |
| **Ethnicity, n (%)** |  |  | 0.931 |
| White Europeans | 8550(93.38) | 2156(94.19) |  |
| Non-White Europeans | 560(6.12) | 125(5.46) |  |
| Missing | 46(0.50) | 8(0.35) |  |
| **BMI (kg/m2)** | 28.49(4.33) | 28.49(4.37) | 0.988 |
| **Employment, n (%)** |  |  | 0.923 |
| Employment | 2540(27.7) | 627(27.39) |  |
| Retired | 5144(56.20) | 1281(55.96) |  |
| Unemployment | 1405(14.40) | 363(15.86) |  |
| Missing | 67(0.73) | 18(0.79) |  |
| **Education, n (%)** |  |  | 0.878 |
| Degree-level education | 661(7.22) | 159(6.59) |  |
| Non-college education | 5357(58.51) | 1349(58.93) |  |
| Missing | 3138(34.27) | 781(34.12) |  |
| **Alcohol consumption status, n (%)** |  |  | 0.581 |
| Never drinkers | 240(2.62) | 53(2.32) |  |
| Current or former drinkers | 8890(97.09) | 2229(97.38) |  |
| Missing | 26(0.28) | 7(0.31) |  |
| **Tobacco consumption status, n (%)** |  |  | 0.567 |
| Never smokers | 1799(19.65) | 446(19.48) |  |
| Current or former smokers | 7287(79.59) | 1829(79.90) |  |
| Missing | 70(0.76) | 14(0.61) |  |
| **Healthy diet score, n (%)** |  |  | 0.829 |
| 0–1 | 1282(14.00) | 317(13.85) |  |
| 2–3 | 5773(63.05) | 1433(62.20) |  |
| 4–5 | 2101(22.95) | 539(23.55) |  |
| **Physical activity, n (%)** |  |  | 0.945 |
| Never | 508(5.55) | 133(5.81) |  |
| Low activity | 377(4.12) | 91(3.98) |  |
| Medium activity | 7237(79.04) | 1802(78.72) |  |
| High activity | 940(10.27) | 236(10.31) |  |
| Missing | 94(1.03) | 27(1.18) |  |
| **TDI** | -1.09(3.17) | -1.10(3.14) | 0.890 |
| **Family income, n (%)** |  |  | 0.945 |
| <£31 000 | 5398(59.00) | 1360(59.41) |  |
| ≥£31 000 | 2356(25.70) | 577(25.21) |  |
| Missing | 1402(15.30) | 32(15.38) |  |
| **SBP (mmHg)** | 144.26(18.24) | 144.29(18.70) | 0.956 |
| **HbA1c (%)** | 5.56(0.61) | 5.58(0.62) | 0.382 |
| **HDL cholesterol (mmol/L)** | 1.24(0.30) | 1.23(0.32) | 0.718 |
| **LDL cholesterol (mmol/L)** | 3.42(0.95) | 3.42(0.95) | 0.918 |
| **Triglycerides (mmol/L)** | 2.09(1.12) | 2.10(1.17) | 0.775 |
| **Cholesterol (mmol/L)** | 5.39(1.26) | 5.39(1.25) | 0.902 |
| **Anti-hypertensive medication, n (%)** | 3519(38.43) | 895(39.10) | 0.574 |
| **Anti-hyperglycaemic medication, n (%)** | 358(3.91) | 87(3.80) | 0.675 |
| **Lipid-lowering medication, n (%)** | 3674(40.13) | 923(40.32) | 0.883 |
| **Physical frailty, n (%)** |  |  |  |
| Non-frail | 4162(45.46) | 945(41.28) | <0.001 |
| Prefrail | 4533(49.51) | 1190(51.99) |  |
| Frail | 461(5.03) | 154(6.73) |  |
| Continuous(per 0.1 unit) | 0.78(0.88) | 0.89(0.94) | <0.001 |
| **Frailty index, n (%)** |  |  |  |
| Non-frail | 4325(47.24) | 881(38.49) | <0.001 |
| Prefrail | 3963(43.28) | 1112(48.58) |  |
| Frail | 868(9.48) | 296(12.93) |  |
| Continuous(per 0.1 unit) | 1.62(0.65) | 1.74(0.67) | <0.001 |

Levels of significance: *P* <0.05 (ANOVA, Kruskal–Wallis H, and chi-squared tests).

Abbreviations: BMI, body mass index; TDI, Townsend deprivation index; SBP, systolic blood pressure; HbA1c, haemoglobin A1c.

**Table S14. Comparison of Baseline Characteristics Between Groups with and without TAA After Propensity Score Matching (1:1)**

| **Baseline Characteristics** | **No TAA**  **(N = 1114)** | **TAA**  **(N =1114)** | ***P*-value** |
| --- | --- | --- | --- |
| **Age (years)** | 60.17(6.99) | 60.22(6.83) | 0.864 |
| **Female sex, n (%)** | 335(30.07) | 351(31.51) | 0.491 |
| **Ethnicity, n (%)** |  |  | 0.458 |
| White Europeans | 1013(90.93) | 1029(92.37) |  |
| Non-White Europeans | 97(8.71) | 81(7.27) |  |
| Missing | 4(0.36) | 4(0.36) |  |
| **BMI (kg/m2)** | 27.89(4.47) | 27.87(4.49) | 0.912 |
| **Employment, n (%)** |  |  | 0.953 |
| Employment | 467(41.90) | 455(40.80) |  |
| Retired | 466(41.80) | 479(43.00) |  |
| Unemployment | 170(15.30) | 169(15.20) |  |
| Missing | 11(0.99) | 11(1.00) |  |
| **Education, n (%)** |  |  | 0.810 |
| Degree-level education | 128(11.49) | 137(12.30) |  |
| Non-college education | 751(67.41) | 739(66.34) |  |
| Missing | 235(21.10) | 238(21.36) |  |
| **Alcohol consumption status, n (%)** |  |  | 0.424 |
| Never drinkers | 31(2.78) | 42(3.77) |  |
| Current or former drinkers | 1082(97.13) | 1071(96.14) |  |
| Missing | 1(0.09) | 1(0.09) |  |
| **Tobacco consumption status, n (%)** |  |  | 0.519 |
| Never smokers | 540(48.47) | 538(48.29) |  |
| Current or former smokers | 569(51.08) | 574(51.53) |  |
| Missing | 5(0.45) | 2(0.18) |  |
| **Healthy diet score, n (%)** |  |  | 0.666 |
| 0–1 | 140(12.57) | 127(11.40) |  |
| 2–3 | 691(62.03) | 694(62.30) |  |
| 4–5 | 283(25.40) | 293(26.30) |  |
| **Physical activity, n (%)** |  |  | 0.796 |
| Never | 58(5.21) | 68(6.10) |  |
| Low activity | 55(4.94) | 49(4.40) |  |
| Medium activity | 874(78.46) | 860(77.20) |  |
| High activity | 114(10.23) | 124(11.13) |  |
| Missing | 13(1.17) | 13(1.17) |  |
| **TDI** | -1.44(3.07) | -1.40(2.96) | 0.788 |
| **Family income, n (%)** |  |  | 0.282 |
| <£31 000 | 481(43.18) | 516(46.32) |  |
| ≥£31 000 | 487(43.72) | 452(40.57) |  |
| Missing | 146(13.11) | 146(13.11) |  |
| **SBP (mmHg)** | 141.52(18.77) | 140.97(18.42) | 0.483 |
| **HbA1c (%)** | 5.57(0.61) | 5.56(0.62) | 0.779 |
| **HDL cholesterol (mmol/L)** | 1.38(0.38) | 1.38(0.37) | 0.644 |
| **LDL cholesterol (mmol/L)** | 3.48(0.90) | 3.46(0.81) | 0.516 |
| **Triglycerides (mmol/L)** | 1.83(1.02) | 1.82(1.02) | 0.784 |
| **Cholesterol (mmol/L)** | 5.55(1.18) | 5.53(1.06) | 0.639 |
| **Anti-hypertensive medication, n (%)** | 283(25.40) | 296(26.57) | 0.562 |
| **Anti-hyperglycaemic medication, n (%)** | 42(3.77) | 38(3.41) | 0.568 |
| **Lipid-lowering medication, n (%)** | 201(18.04) | 210(18.85) | 0.532 |
| **Physical frailty, n (%)** |  |  |  |
| Non-frail | 569(51.08) | 529(47.49) | 0.237 |
| Prefrail | 510(45.78) | 548(49.19) |  |
| Frail | 35(3.14) | 37(3.32) |  |
| Continuous(per 0.1 unit) | 0.67(0.82) | 0.72(0.82) | 0.196 |
| **Frailty index, n (%)** |  |  |  |
| Non-frail | 692(62.10) | 563(50.54) | <0.001 |
| Prefrail | 355(31.90) | 469(42.10) |  |
| Frail | 67(6.00) | 82(7.36) |  |
| Continuous(per 0.1 unit) | 1.44(0.61) | 1.57(0.63) | <0.001 |

Levels of significance: *P* <0.05 (ANOVA, Kruskal–Wallis H, and chi-squared tests).

Abbreviations: BMI, body mass index; TDI, Townsend deprivation index; SBP, systolic blood pressure; HbA1c, haemoglobin A1c.

**Table S15. Comparison of Baseline Characteristics Between Groups with and without TAA After Propensity Score Matching (1:4)**

| **Baseline Characteristics** | **No TAA**  **(N = 4456)** | **TAA**  **(N = 1114)** | ***P*-value** |
| --- | --- | --- | --- |
| **Age (years)** | 60.36(6.78) | 60.22(6.83) | 0.527 |
| **Female sex, n (%)** | 1377(30.90) | 351(31.51) | 0.896 |
| **Ethnicity, n (%)** |  |  | 0.807 |
| White Europeans | 4092(91.83) | 1029(92.37) |  |
| Non-White Europeans | 344(7.72) | 81(7.27) |  |
| Missing | 20(0.45) | 4(0.36) |  |
| **BMI (kg/m2)** | 27.80(4.52) | 27.87(4.49) | 0.613 |
| **Employment, n (%)** |  |  | 0.922 |
| Employment | 1812(40.66) | 455(40.80) |  |
| Retired | 1954(43.85) | 479(43.00) |  |
| Unemployment | 644(14.45) | 169(15.20) |  |
| Missing | 46(1.03) | 11(1.00) |  |
| **Education, n (%)** |  |  | 0.729 |
| Degree-level education | 510(11.45) | 137(12.30) |  |
| Non-college education | 2985(66.99) | 739(66.34) |  |
| Missing | 961(21.57) | 238(21.36) |  |
| **Alcohol consumption status, n (%)** |  |  | 0.490 |
| Never drinkers | 153(3.43) | 42(3.77) |  |
| Current or former drinkers | 4302(96.54) | 1071(96.14) |  |
| Missing | 1(0.03) | 1(0.09) |  |
| **Tobacco consumption status, n (%)** |  |  | 0.917 |
| Never smokers | 2152(48.29) | 538(48.29) |  |
| Current or former smokers | 2293(51.46) | 574(51.53) |  |
| Missing | 11(0.25) | 2(0.18) |  |
| **Healthy diet score, n (%)** |  |  | 0.825 |
| 0–1 | 502(11.27) | 127(11.40) |  |
| 2–3 | 2819(63.26) | 694(62.30) |  |
| 4–5 | 1135(25.47) | 293(26.30) |  |
| **Physical activity, n (%)** |  |  | 0.943 |
| Never | 250(5.61) | 68(6.10) |  |
| Low activity | 205(4.60) | 49(4.40) |  |
| Medium activity | 3477(78.00) | 860(77.20) |  |
| High activity | 473(10.60) | 124(11.13) |  |
| Missing | 51(1.14) | 13(1.17) |  |
| **TDI** | -1.42(3.06) | -1.40(2.96) | 0.899 |
| **Family income, n (%)** |  |  | 0.982 |
| <£31 000 | 2050(46.10) | 516(46.32) |  |
| ≥£31 000 | 1817(40.78) | 452(40.57) |  |
| Missing | 589(13.22) | 146(13.11) |  |
| **SBP (mmHg)** | 141.59(18.50) | 140.97(18.42) | 0.314 |
| **HbA1c (%)** | 5.56(0.61) | 5.56(0.62) | 0.799 |
| **HDL cholesterol (mmol/L)** | 1.39(0.37) | 1.38(0.37) | 0.875 |
| **LDL cholesterol (mmol/L)** | 3.48(0.95) | 3.46(0.81) | 0.469 |
| **Triglycerides (mmol/L)** | 1.84(1.05) | 1.82(1.02) | 0.663 |
| **Cholesterol (mmol/L)** | 5.56(1.16) | 5.53(1.06) | 0.487 |
| **Anti-hypertensive medication, n (%)** | 1125(25.25) | 296(26.57) | 0.385 |
| **Anti-hyperglycaemic medication, n (%)** | 157(3.52) | 38(3.41) | 0.546 |
| **Lipid-lowering medication, n (%)** | 814(18.27) | 210(18.85) | 0.412 |
| **Physical frailty, n (%)** |  |  |  |
| Non-frail | 2264(50.81) | 529(47.49) | 0.081 |
| Prefrail | 2026(45.47) | 548(49.19) |  |
| Frail | 166(3.73) | 37(3.32) |  |
| Continuous(per 0.1 unit) | 0.68(0.83) | 0.72(0.82) | 0.184 |
| **Frailty index, n (%)** |  |  |  |
| Non-frail | 2615(58.68) | 563(50.54) | <0.001 |
| Prefrail | 1569(35.21) | 469(42.10) |  |
| Frail | 272(6.10) | 82(7.36) |  |
| Continuous(per 0.1 unit) | 1.47(0.61) | 1.57(0.63) | <0.001 |

Levels of significance: *P* <0.05 (ANOVA, Kruskal–Wallis H, and chi-squared tests).

Abbreviations: BMI, body mass index; TDI, Townsend deprivation index; SBP, systolic blood pressure; HbA1c, haemoglobin A1c.

**Table S16. Comparison of Baseline Characteristics Between Groups with and without AD After Propensity Score Matching (1:1)**

| **Baseline Characteristics** | **No AD**  **(N = 310)** | **AD**  **(N = 310)** | ***P*-value** |
| --- | --- | --- | --- |
| **Age (years)** | 60.69(6.74) | 60.37(7.05) | 0.560 |
| **Female sex, n (%)** | 116(37.42) | 122(39.35) | 0.680 |
| **Ethnicity, n (%)** |  |  | 0.632 |
| White Europeans | 276(89.03) | 283(91.29) |  |
| Non-White Europeans | 33(10.65) | 26(8.39) |  |
| Missing | 1(0.32) | 1(0.32) |  |
| **BMI (kg/m2)** | 27.92(4.74) | 27.88(4.58) | 0.916 |
| **Employment, n (%)** |  |  | 0.469 |
| Employment | 127(40.79) | 120(38.71) |  |
| Retired | 140(45.16) | 135(43.55) |  |
| Unemployment | 43(13.87) | 54(17.42) |  |
| Missing | 0(0) | 1(0.32) |  |
| **Education, n (%)** |  |  | 0.701 |
| Degree-level education | 34(10.97) | 35(11.29) |  |
| Non-college education | 196(63.23) | 204(65.81) |  |
| Missing | 80(25.81) | 71(22.90) |  |
| **Alcohol consumption status, n (%)** |  |  | 0.999 |
| Never drinkers | 12(3.87) | 13(4.19) |  |
| Current or former drinkers | 298(96.13) | 297(95.81) |  |
| Missing | 0(0) | 0(0) |  |
| **Tobacco consumption status, n (%)** |  |  | 0.423 |
| Never smokers | 122(39.35) | 136(43.87) |  |
| Current or former smokers | 187(60.32) | 172(55.48) |  |
| Missing | 1(0.32) | 2(0.65) |  |
| **Healthy diet score, n (%)** |  |  | 0.312 |
| 0–1 | 29(9.35) | 41(13.23) |  |
| 2–3 | 192(61.94) | 185(59.68) |  |
| 4–5 | 89(28.71) | 84(27.10) |  |
| **Physical activity, n (%)** |  |  | 0.922 |
| Never | 15(4.84) | 12(3.87) |  |
| Low activity | 14(4.52) | 11(3.55) |  |
| Medium activity | 239(77.10) | 247(79.70) |  |
| High activity | 35(11.30) | 34(11.00) |  |
| Missing | 7(2.26) | 6(1.94) |  |
| **TDI** | -0.68(3.56) | -1.13(3.24) | 0.097 |
| **Family income, n (%)** |  |  | 0.437 |
| <£31 000 | 164(52.90) | 148(47.74) |  |
| ≥£31 000 | 106(34.19) | 117(37.74) |  |
| Missing | 40(12.90) | 45(14.52) |  |
| **SBP (mmHg)** | 142.98(18.16) | 144.34(19.01) | 0.368 |
| **HbA1c (%)** | 5.59(0.64) | 5.57(0.60) | 0.345 |
| **HDL cholesterol (mmol/L)** | 1.38(0.37) | 1.40(0.37) | 0.652 |
| **LDL cholesterol (mmol/L)** | 3.48(0.83) | 3.47(0.86) | 0.887 |
| **Triglycerides (mmol/L)** | 1.81(0.91) | 1.77(1.01) | 0.598 |
| **Cholesterol (mmol/L)** | 5.51(1.09) | 5.53(1.15) | 0.802 |
| **Anti-hypertensive medication, n (%)** | 75(24.19) | 69(22.26) | 0.634 |
| **Anti-hyperglycaemic medication, n (%)** | 3(0.97) | 2(0.65) | 0.999 |
| **Lipid-lowering medication, n (%)** | 67(21.61) | 58(18.71) | 0.423 |
| **Physical frailty, n (%)** |  |  |  |
| Non-frail | 143(46.13) | 158(50.97) | 0.315 |
| Prefrail | 150(48.39) | 141(45.48) |  |
| Frail | 17(5.48) | 11(3.55) |  |
| Continuous(per 0.1 unit) | 0.80(0.90) | 0.70(0.86) | 0.188 |
| **Frailty index, n (%)** |  |  |  |
| Non-frail | 162(52.26) | 164(52.90) | 0.908 |
| Prefrail | 121(39.03) | 122(39.35) |  |
| Frail | 27(8.71) | 24(7.74) |  |
| Continuous(per 0.1 unit) | 1.56(0.65) | 1.55(0.64) | 0.755 |

Levels of significance: *P* <0.05 (ANOVA, Kruskal–Wallis H, and chi-squared tests).

Abbreviations: BMI, body mass index; TDI, Townsend deprivation index; SBP, systolic blood pressure; HbA1c, haemoglobin A1c.

**Table S17. Comparison of Baseline Characteristics Between Groups with and without AD After Propensity Score Matching (1:4)**

| **Baseline Characteristics** | **No AD**  **(N = 1240)** | **AD**  **(N = 310)** | ***P*-value** |
| --- | --- | --- | --- |
| **Age (years)** | 60.60(6.65) | 60.37(7.05) | 0.996 |
| **Female sex, n (%)** | 493(39.76) | 122(39.35) | 0.820 |
| **Ethnicity, n (%)** |  |  | 0.931 |
| White Europeans | 1124(90.65) | 283(91.29) |  |
| Non-White Europeans | 111(8.95) | 26(8.39) |  |
| Missing | 5(0.40) | 1(0.32) |  |
| **BMI (kg/m2)** | 27.78(4.75) | 27.88(4.58) | 0.943 |
| **Employment, n (%)** |  |  | 0.701 |
| Employment | 502(40.48) | 120(38.71) |  |
| Retired | 526(42.42) | 135(43.55) |  |
| Unemployment | 211(17.02) | 54(17.42) |  |
| Missing | 1(0.08) | 1(0.32) |  |
| **Education, n (%)** |  |  | 0.983 |
| Degree-level education | 141(11.37) | 35(11.29) |  |
| Non-college education | 821(66.21) | 204(65.81) |  |
| Missing | 278(22.42) | 71(22.90) |  |
| **Alcohol consumption status, n (%)** |  |  | 0.581 |
| Never drinkers | 39(3.15) | 13(4.19) |  |
| Current or former drinkers | 1200(96.77) | 297(95.81) |  |
| Missing | 1(0.08) | 0(0) |  |
| **Tobacco consumption status, n (%)** |  |  | 0.977 |
| Never smokers | 539(43.37) | 136(43.87) |  |
| Current or former smokers | 694(55.97) | 172(55.48) |  |
| Missing | 7(0.56) | 2(0.65) |  |
| **Healthy diet score, n (%)** |  |  | 0.689 |
| 0–1 | 148(11.94) | 41(13.23) |  |
| 2–3 | 730(58.87) | 185(59.68) |  |
| 4–5 | 362(29.19) | 84(27.10) |  |
| **Physical activity, n (%)** |  |  | 0.973 |
| Never | 56(4.52) | 12(3.87) |  |
| Low activity | 47(3.79) | 11(3.55) |  |
| Medium activity | 976(78.71) | 247(79.70) |  |
| High activity | 132(10.65) | 34(11.00) |  |
| Missing | 29(2.34) | 6(1.94) |  |
| **TDI** | -1.01(3.34) | -1.13(3.24) | 0.914 |
| **Family income, n (%)** |  |  | 0.973 |
| <£31 000 | 582(46.94) | 148(47.74) |  |
| ≥£31 000 | 481(38.79) | 117(37.74) |  |
| Missing | 177(14.27) | 45(14.52) |  |
| **SBP (mmHg)** | 144.19(19.15) | 144.34(19.01) | 0.883 |
| **HbA1c (%)** | 5.58(0.61) | 5.57(0.60) | 0.804 |
| **HDL cholesterol (mmol/L)** | 1.40(0.38) | 1.40(0.37) | 0.774 |
| **LDL cholesterol (mmol/L)** | 3.52(0.85) | 3.47(0.86) | 0.770 |
| **Triglycerides (mmol/L)** | 1.78(0.91) | 1.77(1.01) | 0.856 |
| **Cholesterol (mmol/L)** | 5.61(1.12) | 5.53(1.15) | 0.665 |
| **Anti-hypertensive medication, n (%)** | 258(20.81) | 69(22.26) | 0.629 |
| **Anti-hyperglycaemic medication, n (%)** | 9(0.73) | 2(0.65) | 0.999 |
| **Lipid-lowering medication, n (%)** | 231(18.63) | 58(18.71) | 0.998 |
| **Physical frailty, n (%)** |  |  |  |
| Non-frail | 633(51.05) | 158(50.97) | 0.868 |
| Prefrail | 555(44.76) | 141(45.48) |  |
| Frail | 52(4.19) | 11(3.55) |  |
| Continuous(per 0.1 unit) | 0.70(0.85) | 0.70(0.86) | <0.001 |
| **Frailty index, n (%)** |  |  |  |
| Non-frail | 677(54.60) | 164(52.90) | 0.866 |
| Prefrail | 470(37.90) | 122(39.35) |  |
| Frail | 93(7.50) | 24(7.74) |  |
| Continuous(per 0.1 unit) | 1.53(0.63) | 1.55(0.64) | 0.631 |

Levels of significance: *P* <0.05 (ANOVA, Kruskal–Wallis H, and chi-squared tests).

Abbreviations: BMI, body mass index; TDI, Townsend deprivation index; SBP, systolic blood pressure; HbA1c, haemoglobin A1c.

**Table S18. Association of frailty status with incident AA and AD After Propensity Score Matching (1:4).**

|  | **HR (95% CI)** | ***P* value** |
| --- | --- | --- |
| **AA** |  |  |
| Physical frailty |  |  |
| Non-frail | 1.00 (Ref) |  |
| Prefrail | 1.12 (1.05-1.20) | <0.001 |
| Frail | 1.30 (1.12-1.50) | <0.001 |
| Continuous(per 0.1 unit) | 1.10 (1.06-1.14) | <0.001 |
| Frailty index |  |  |
| Non-frail | 1.00 (Ref) |  |
| Prefrail | 1.28 (1.20-1.37) | <0.001 |
| Frail | 1.47(1.32-1.64) | <0.001 |
| Continuous(per 0.1 unit) | 1.24 (1.18-1.30) | <0.001 |
| **AAA** |  |  |
| Physical frailty |  |  |
| Non-frail | 1.00 (Ref) |  |
| Prefrail | 1.15 (1.06-1.26) | <0.001 |
| Frail | 1.43 (1.21-1.70) | <0.001 |
| Continuous(per 0.1 unit) | 1.13 (1.09-1.18) | <0.001 |
| Frailty index |  |  |
| Non-frail | 1.00 (Ref) |  |
| Prefrail | 1.34 (1.23-1.47) | <0.001 |
| Frail | 1.60 (1.40-1.82) | <0.001 |
| Continuous(per 0.1 unit) | 1.29 (1.21-1.37) | <0.001 |
| **TAA** |  |  |
| Physical frailty |  |  |
| Non-frail | 1.00 (Ref) |  |
| Prefrail | 1.16 (1.03-1.30) | 0.018 |
| Frail | 0.97 (0.69-1.35) | 0.838 |
| Continuous(per 0.1 unit) | 1.05 (0.98-1.13) | 0.134 |
| Frailty index |  |  |
| Non-frail | 1.00 (Ref) |  |
| Prefrail | 1.35 (1.19-1.52) | <0.001 |
| Frail | 1.37 (1.09-1.73) | 0.008 |
| Continuous(per 0.1 unit) | 1.24 (1.14-1.36) | <0.001 |
| **AD** |  |  |
| Physical frailty |  |  |
| Non-frail | 1.00 (Ref) |  |
| Prefrail | 1.02 (0.81-1.28) | 0.878 |
| Frail | 0.87 (0.47-1.61) | 0.663 |
| Continuous(per 0.1 unit) | 1.01 (0.89-1.15) | 0.846 |
| Frailty index |  |  |
| Non-frail | 1.00 (Ref) |  |
| Prefrail | 1.09 (0.87-1.38) | 0.451 |
| Frail | 1.13 (0.74-1.74) | 0.565 |
| Continuous(per 0.1 unit) | 1.08 (0.91-1.28) | 0.401 |

Levels of significance: *P* <0.05 (Cox regression model).

Abbreviations: HR, hazard ratio; AA, aortic aneurysm; AAA, abdominal aortic aneurysm; TAA, thoracic aortic aneurysm.

Cox regression model was adjusted for age, sex, ethnicity, educational level, employment status, family income, TDI, alcohol and tobacco consumption, healthy diet score, physical activity, BMI, SBP, HbA1c, lipid profile and the use of anti-hypertensive medication, anti-hyperglycaemic medication, and lipid-lowering medications.

**Figure S1. Cumulative risks of incident AAA, TAA, AD in accordance with frailty profile.** Levels of significance: *P* <0.05 (log-rank test). Abbreviations: AAA, abdominal aortic aneurysm; TAA, thoracic aortic aneurysm.

**Figure S2. Dose-response association of frailty with the risks of incident AAA, TAA and AD.** Dark lines represent hazard ratios, and shaded areas represent 95% CIs. Data were fitted by using the restricted cubic spline with three knots. Models were adjusted for age, sex, ethnicity, educational level, employment status, family income, Townsend deprivation index, alcohol and tobacco consumption, healthy diet score, physical activity, body mass index, systolic blood pressure, glycated haemoglobin, lipid profile and the use of anti-hypertensive medication, anti-hyperglycaemic medication, and lipid-lowering medications. Levels of significance: *P* <0.05. Abbreviations: AAA, abdominal aortic aneurysm; TAA, thoracic aortic aneurysm.

**Figure S3. Stratified analysis for the association between frailty status and AAA risk.** Subgroup analyses were conducted for prefrailty and frailty compared with non-frailty. Multivariable Cox models were adjusted for age, sex, ethnicity, educational level, employment status, family income, Townsend deprivation index, alcohol and tobacco consumption, healthy diet score, physical activity, body mass index, systolic blood pressure, glycated haemoglobin, lipid profile and the use of anti-hypertensive medication, anti-hyperglycaemic medication, and lipid-lowering medications. Levels of significance: *P* <0.05. Abbreviations: HR, hazard ratio; AAA, abdominal aortic aneurysm.

**Figure S4. Stratified analysis for the association between frailty status and TAA risk.** Subgroup analyses were conducted for prefrailty and frailty compared with non-frailty. Multivariable Cox models were adjusted for age, sex, ethnicity, educational level, employment status, family income, Townsend deprivation index, alcohol and tobacco consumption, healthy diet score, physical activity, body mass index, systolic blood pressure, glycated haemoglobin, lipid profile and the use of anti-hypertensive medication, anti-hyperglycaemic medication, and lipid-lowering medications. Levels of significance: *P* <0.05. Abbreviations: HR, hazard ratio; TAA, thoracic aortic aneurysm.

**Figure S5. Stratified analysis for the association between frailty status and AD risk.** Subgroup analyses were conducted for prefrailty and frailty compared with non-frailty. Multivariable Cox models were adjusted for age, sex, ethnicity, educational level, employment status, family income,Townsend deprivation index, alcohol and tobacco consumption, healthy diet score, physical activity, body mass index, systolic blood pressure, glycated haemoglobin, lipid profile and the use of anti-hypertensive medication, anti-hyperglycaemic medication, and lipid-lowering medications. Levels of significance: *P* <0.05. Abbreviations: HR, hazard ratio; AD, aortic dissection.
